# Colloidal Deacetylation of Chitin Nanocrystals Results in Amorphous and Patchy Chitosan Chains

**DOI:** 10.1021/acsnano.5c21467

**Published:** 2026-04-10

**Authors:** Tony Jin, Saskia Heermant, Hanieh Mianehrow, Thomas G. Parton, Antonio Carone, Ruslan Nedielkov, Yu Ogawa, Jacek Kozuch, Silvia Vignolini

**Affiliations:** † Department of Sustainable and Bio-Inspired Materials, 28321 Max Planck Institute of Colloids and Interfaces, Am Muhlenberg 1, 14476 Potsdam, Germany; ‡ Experimental Molecular Biophysics, Department of Physics, 9166 Freie Universität Berlin, Arnimallee 14, 14195 Berlin, Germany; § Department of Chemistry, 26583 University of Potsdam, Karl-Liebknecht Straße 24-25, 14476 Potsdam, Germany; ∥ Univ. Grenoble Alpes, CNRS, CERMAV, 38000 Grenoble, France; ⊥ Physical Chemistry of Biomolecular Systems, Institute of Physical Chemistry and Theoretical Chemistry, BRICS − Braunschweig Integrated Centre of Systems Biology, Rebenring 56, 38106 Braunschweig, Germany

**Keywords:** chitin, chitosan, nanocrystals, electron diffraction, scanning near-field optical microscopy

## Abstract

Controlling the surface chemistry of biobased nanomaterials is crucial for unlocking their full potential in advanced applications. However, the impact of such chemical modifications on nanoscale morphology remains poorly understood. In this work, we investigate the sequential deacetylation of chitin nanocrystals (ChNCs) into chitosan nanocrystals (ChsNCs), a transformation that significantly alters their ultrastructural properties through the introduction of amine functionalities. By combining bulk and nanoscale characterization techniques—including electron diffraction, cryo-transmission electron microscopy (cryoTEM), and scattering-type scanning near-field optical microscopy (s-SNOM)—we can track the chemical and structural evolution during the deacetylation process. Our findings demonstrate that partially deacetylated ChNCs (20–60% degree of deacetylation) exhibit chitosan-rich surface patches, revealing nanoscale heterogeneity in surface modification. Furthermore, we observed that such a patchy distribution is accompanied by a decrease in nanocrystal bundling, suggesting changes in interparticle interactions. Finally, at higher degrees of deacetylation, ChsNCs exhibit mobile chitosan chains surrounding cores composed of chitosan-rich or residual chitin regions. We believe that our results provide critical insights into the nanostructural identity of ChsNCs, with implications for understanding and tuning their structure–property–function relationships, which are critical for the fabrication of chitin-derived biomaterials.

## Graphical abstract

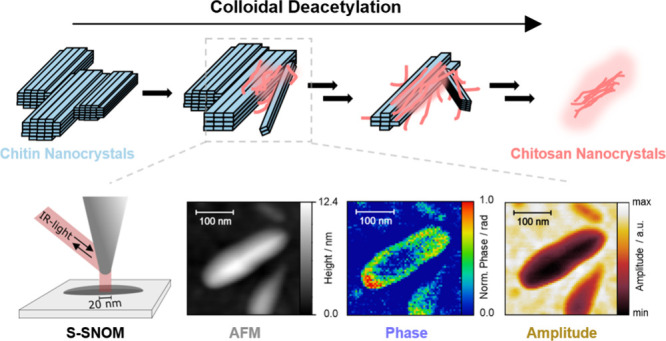

Biobased polymers such as chitin, composed of β-(1→4)-linked *N*-acetyl-d-glucosamine, and cellulose, composed of β-(1→4)-linked d-glucose, have emerged as versatile building blocks for bioderived soft nanomaterials, offering unique mechanical, optical, and interfacial properties.^1,2^ Through controlled hydrolysis, chitin nanocrystals (ChNCs) and cellulose nanocrystals (CNCs) can be liberated from their native microfibrillar form.^3,4^ While both nanocrystalline forms share similar rod-like morphology and crystallinity, ChNCs stand out due to the presence of nitrogen in the form of mostly acetamide groups with a low population of primary amine groups, quantified using the degree of deacetylation (DDA), with ChNCs having a DDA of around 5–15%.^5^ This allows ChNCs to have a positive surface charge under acidic conditions and greater potential for surface modifications, distinguishing them from their cellulose-based counterparts and expanding their range of applications.^6–8^

In recent years, there has been growing interest in the functionalization of extracted ChNC via deacetylation to produce chitosan nanocrystals (ChsNCs), aiming to introduce even more primary amine groups to augment optical and structural properties while retaining the rod-like morphology.^9,10^ Early efforts, including those by Hsieh and co-workers,^11^ achieved only partial deacetylation, typically up to ∼50% DDA, while preserving the rod-like morphology. In other cases, researchers were able to reach higher DDA values, but often at the expense of nanocrystal integrity, resulting in loss of crystallinity and destruction of the rod-like structure.^12^ However, recent studies have successfully produced ChsNCs with high DDA without compromising the rod-like morphology of the nanocrystals, utilizing NaBH_4_ as a reducing agent during the process.^13^ These nanocrystals had superior capabilities in areas such as nanoadhesion,^14^ hydrogels,^15^ and catalyst supports,^16^ highlighting the powerful role of surface modification in tuning polysaccharide nanocrystal behavior.

Despite these promising advances, the ultrastructural organization of highly deacetylated ChsNCs has remained poorly understood, with previous reports only showing ensemble-averaged analyses^13,17^ or morphological information on nondeacetylated ChNCs.^10^ In the context of bulk or macromolecular chitin, recent studies have reported the presence of a deacetylation behavior,^18^ which is known to exhibit distinct biological activity compared to randomly deacetylated analogs.^19^ There has been no attempt to acquire better understanding of the deacetylation event at the nano and molecular-scale level.

Here, we study how and where the deacetylation reaction proceeds, and its distribution and uniformity within individual nanocrystals. Specifically, we reveal the nanoscale heterogeneity of deacetylation within single ChNCs, demonstrating that surface modification is not uniform, but spatially patterned. We apply a multimodal ultrastructural analysis combining scattering-type scanning near-field optical microscopy (s-SNOM), nanoscale Fourier transform infrared spectroscopy (nano-FTIR), cryo-electron microscopy (cryo-EM), and electron diffraction, alongside molecular dynamics simulations, to directly probe the deacetylation process within individual nanocrystals and uncover the spatial organization of chemical transformation at the nanoscale. To our knowledge, this is the first attempt at providing a unified morphological and chemical structure of deacetylated ChNCs. With these findings, we believe it can lead to more precise refinement and spatial control of surface functionalization of polysaccharide nanomaterials.

## Results/Discussion

### Deacetylation Occurs throughout the Nanocrystal Bulk

The deacetylation reaction of bulk polymeric chitin is usually defined as a heterogeneous reaction in which *N*-acetylglucosamine (NAG) units are deacetylated into glucosamine in a pseudo-first-order reaction in excess base.^20^ Chemically, it is defined as an S_N_2 nucleophilic attack from the hydroxide anion toward the carbamide carbon, forming a dihydroxy intermediate (Scheme 1). While this step is reversible, the transfer of the proton from the hydroxy to another free hydroxide forms a dianion intermediate, which decomposes toward an acetylate anion and the primary amine found on the liberated glucosamine unit. It is mostly termed heterogeneous as polymeric chitin is insoluble under the reaction conditions, and swelling of the chitin powder within the alkali solution allows deacetylation to proceed past the surface of the particulates, known as the “shrinking core” model.^21,22^

**Scheme 1:**
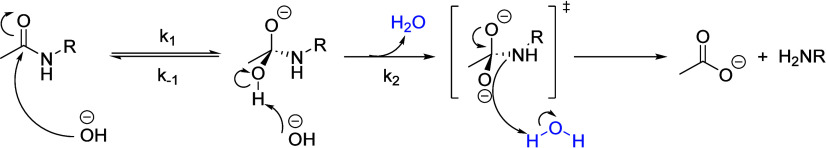
General Deacetylation Mechanism in the Presence of Excess Base

In contrast to the bulk case, observations of deacetylation of colloidal particles suggest a more complex progression. Previous works have demonstrated that carboxylated ChNCs made using ammonium persulfate as a mild oxidizing agent can be sequentially deacetylated with NaBH_4_, while maintaining rod-like morphology, which we are denoting as “sequential deacetylation” in this article. Under basic conditions, NaBH_4_ reduces the reducing ends of chitin chains, which preferentially form aldehydes.^13^ This inhibits the “end-peeling” process commonly associated with wood pulping, in which concentrated base reflux conditions hydrolyze polysaccharides into short-chain oligomers and monosaccharides.^23^

Using this procedure, it was realized that deacetylation, even over an 18 h reaction time, led to a relatively low increase in DDA.^13^ This self-limiting reaction can be attributed to the theories on macromolecular deacetylation, where deacetylation is also limited. The two major theories are based on chitin hydration and a quasi-stable negatively charged amine under extremely alkaline conditions.^20^ In a highly alkaline environment, deacetylation will increase the hydration of chitin particles, while also producing a negatively charged amine intermediate (R-NH^–^), both of which decrease the rate of deacetylation and ultimately halt it. Only when you quench the reaction via dilution do you then negate the properties of hydration while also rapidly protonating the intermediate to its final form (R-NH_2_). In the colloidal context (this work), we also assume these to be the two limiting processes which halt the reaction, although the dominant process is still up to debate, and naturally very interesting to study in future works both experimentally and theoretically.

This observation suggests indirectly that using purely “crystalline” chitin inhibits the deacetylation reaction, which usually undergoes fast deacetylation followed by a slow step up to 60% DDA using bulk macromolecular chitin.^24^ In contrast, sequential deacetylation (i.e., performing multiple deacetylation reactions with washing steps in between) led to the formation of nanocrystals with DDA > 60%, defined as “chitosan” nanocrystals.^13^ However, whatever the approach and the degree of deacetylation achieved, little is known of where the chitosan residues are located on the rod-like colloidal particles. In this work, we produced an initial ChNC suspension by HCl acid hydrolysis, which preserves the crystalline chitin of the source without introducing additional functional groups that would add complexity to the analysis. This approach is therefore preferable to using carboxylated ChNCs, which have additional negatively charged moieties. We performed sequential deacetylation on ChNCs to obtain a set of samples with different degrees of DDA. To label this series, all ChsNC samples will be designated as *x*ChsNC, where *x* denotes the number of deacetylation reactions performed (Figure 1a and Table 1). An interesting observation noted is that the deacetylation takes much longer to reach high levels of DDA (>70%) than in the macromolecular case,^22^ warranting future study in examining the kinetic parameters that affect colloidal deacetylation.

**Table 1:** Summary of the ChsNC Series and Their Resultant Colloidal Properties

**sample**	**ChNC**	**1ChsNC**	**3ChsNC**	**5ChsNC**
deacetylation runs	0	1	3	5
yield (%)	34^a^	53^b^	49^b^	51^b^
DDA from ssNMR (%)	5	20	47	98
DDA from titration (%)	7	22	59	96
accessible amine content from titration (mmol kg^–1^)	381	1231	3281	5343
cryoEM mean length (nm)	160 ± 60	150 ± 60	160 ± 60	190 ± 60
cryoEM mean width (nm)	8 ± 3	9 ± 2	10 ± 3	10 ± 3
*Z*-average (nm)	180 ± 1	190 ± 2	200 ± 1	220 ± 1
PdI	0.21 ± 0.01	0.19 ± 0.01	0.190 ± 0.004	0.15 ± 0.02
ζ-potential (mV)	50 ± 3	41.2 ± 0.9	42.1 ± 0.7	41 ± 2
dry TEM mean length (nm)	310 ± 140	290 ± 110	230 ± 100	190 ± 70
dry TEM area equivalent width (nm)	24 ± 10	22 ± 8	17 ± 6	13 ± 4
rectangularity	0.62 ± 0.11	0.63 ± 0.10	0.66 ± 0.13	0.69 ± 0.08

a Relative to the starting purified chitin

b Relative to the starting ChNC. All ± is standard deviation values.

**Figure 1:**
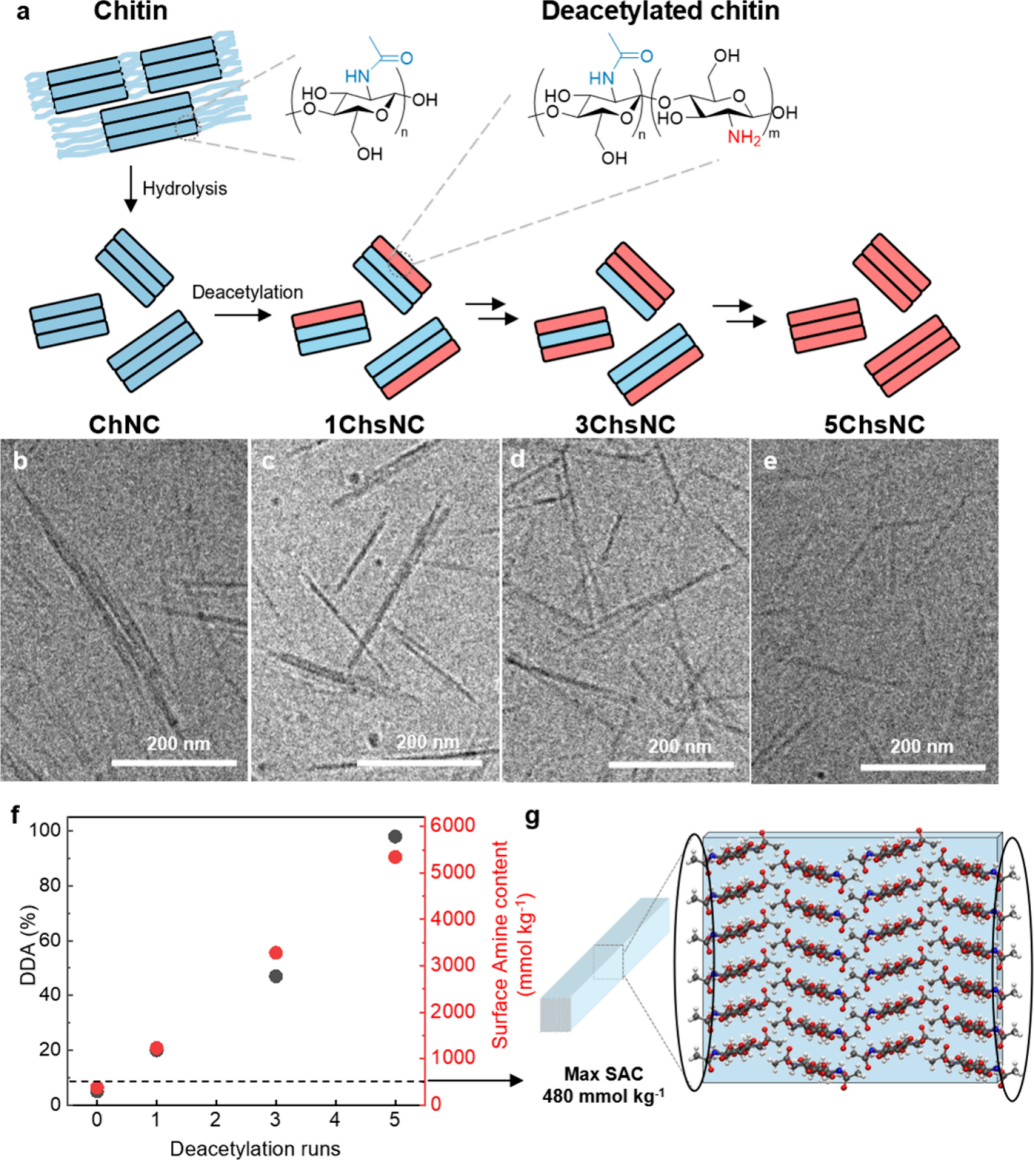
(a) Scheme demonstrating the colloidal deacetylation process. CryoEM micrographs of (b) ChNC, (c) 1ChsNC, (d) 3ChsNC, and (e) 5ChsNC demonstrating the retention of a rod-like morphology. (f) DDA (%, measured by ^13^C ssNMR) and surface amine content (measured by conductometric titration) as a function of the number of deacetylation cycles, with 0 being ChNC. The dashed line indicates the maximum surface amines available on the surface of a ChNC, as modeled utilizing (g) A schematic of the chitin crystal structure.

The cryogenic transmission electron microscopy (cryoTEM) images reported in Figure 1b–e confirm that the nanocrystals found in the series exhibit a rod-like appearance in suspension, demonstrating the retention of a colloidal particle throughout the entire deacetylation process. Notably, we observed that while the length and width of nanocrystals in cryoEM revealed no significant reduction in the dimensions of individual nanocrystals throughout the deacetylation procedure (Table 1). This is also verified through the mean hydrodynamic size (*Z*-average) measurements of the series through dynamic light scattering (DLS), which match closely with measured length values from cryoTEM. The DDA of the as-produced ChsNCs was measured using ^13^C MAS NMR (ssNMR) (Figure S1), confirming full deacetylation starting from 5% for ChNCs up to >95% 5ChsNCs (Table 1, Figure 1f).

Conductometric titration was then employed to analyze the surface amine content (SAC) (Figures 1f and S2) to compare with the “bulk” amine content as found through ssNMR. It is noted that a more accurate interpretation of the amine content calculated from conductometric titrations is rather the quantity of amines accessible to hydroxide neutralization, rather than just the amines at the surface of each colloid. Nevertheless, this approach enables us to differentiate between the total number of amine units present in the sample (as determined by ssNMR) and the accessible amine units, which are presumed to be located at the surface of the nanocrystals. Surprisingly, the surface amine content increases linearly as the nanocrystals are deacetylated. This is further visualized when the surface amine content (mmol kg^–1^) is converted into surface DDA (mol/mol %), which matches the DDA calculated from ssNMR (last row in Table 1, Figure S2). Another ChsNC series was produced to verify these results, which also demonstrates the reproducibility of the sequential deacetylation procedure (Figure S3). Such results suggest that even the more interior core chitosan units may contribute to the total surface charge density of the nanocrystals, and therefore to the electric double-layer of the colloid at pH lower than p*K*_a_ ∼ 6.3 of chitosan. By employing a chitin chain model with a square cross-section^25,26^ and using the length and width measurements from cryoEM, the theoretical maximum for surface amine coverage is calculated to be approximately 480 mmol kg^–1^ (Figure 1g), which is already surpassed after the first deacetylation cycle to create 1ChsNC. This comparison of the DDA at the surface vs bulk gives us insight that all the chitosan units are accessible by hydroxide anions, which negates a simplistic model where a dense core of chitin/chitosan is surrounded by chitosan chains, which are the only ones to provide surface charge for the nanocrystals. Our observations suggest that a more complex organization and distribution of deacetylated units are present in the xCHsNC, which cannot be explained by the simple core–shell model.

### s-SNOM Demonstrates Presence of Chitosan Patches on Individual Nanoparticles

To track the nanoscale composition of individual nanocrystals during deacetylation, we employed IR nanoscopic methods, including nano-FTIR spectroscopy and s-SNOM imaging. Both methods utilize an atomic force microscopy (AFM) tip as an antenna to localize IR radiation at the apex of the tip and provide IR nanoscopic information at a resolution beyond the diffraction limit, given by the tip diameter (Figure 2a).^27–29^ In nano-FTIR spectroscopy, a broad-band IR laser source is used to record local IR absorbance spectra with a spatial resolution of 20 nm. In s-SNOM, a single wavenumber of a tunable quantum cascade laser is selected to obtain corresponding IR nanoscopic maps, along with topographical images from AFM. Previous works on polysaccharide systems utilized s-SNOM and AFM-IR, which provide IR absorbance maps by detecting the photothermal expansion of an AFM tip, to probe nanoscale properties such as amorphous regions in cellulose nanofibers,^30^ and to map the presence of cellulose nanocrystals in polymer composites.^31^ To the best of our knowledge, this is the first case of using s-SNOM toward mapping the surface functionality of chitin-based nanomaterials.

**Figure 2:**
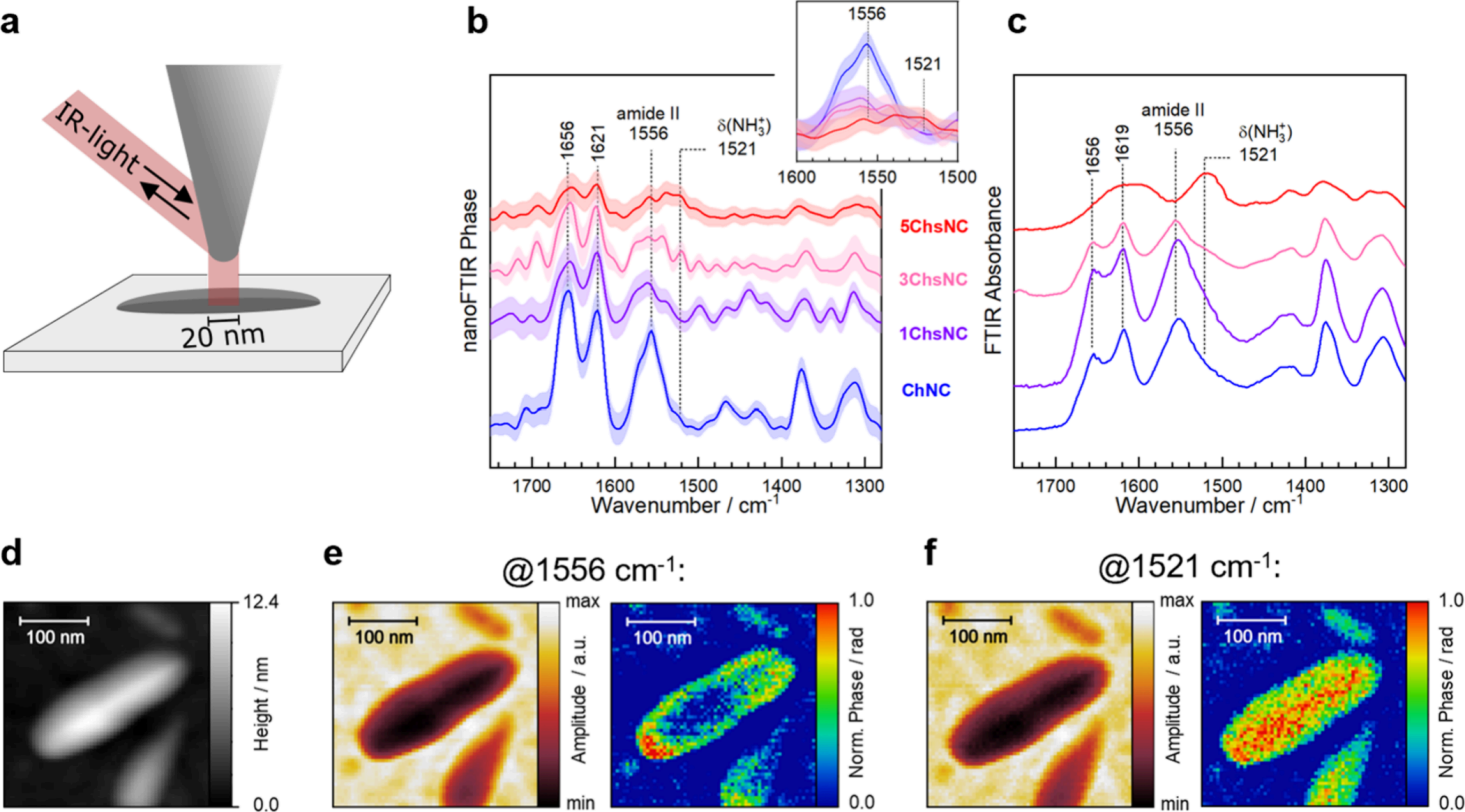
(a) Schematic representation of the s-SNOM setup, demonstrating the usage in topography mode as well as visualizing the amplitude and phase of the IR signal. (b) Nano-FTIR spectra of the xChsNC series averaged over 10 points in comparison with (c) ATR-FTIR spectra of the series demonstrating analogous vibration modes for the amide I and amide II bands of chitin and chitosan. The inset in (b) shows nano-FTIR spectra overlapped, highlighting the sensitivity of the N–H bending amide II at 1556 cm^–1^ for the presence of *N*-acetylglucosamine (chitin) units, and the insensitivity of the δ­(NH_3_^+^) for the detection of glucosamine (chitosan) units. (d) Representative s-SNOM topography image (AFM tapping mode) and its amplitude and phase image at (e) 1556 cm^–1^ and (f) 1521 cm^–1^.

In order to compare the nano-FTIR spectra of the nanocrystals to their corresponding bulk FTIR spectra, we deposited the nanoparticles on templated-stripped Au (TSAu) surfaces (see additional methods section in Supplemental Information) Methods/Experimental Section), which provide an atomically flat substrate as well as a background of no absorbance in the IR range of interest. We then collected spectra from different nanocrystals with different degrees of deacetylation from ChNC to 5ChsNC. Figure 2b reports the averaged nano-FTIR spectra collected from >10 individual nanocrystals (detected at the second harmonic of the tip frequency) for samples with different degrees of deacetylation. In such spectra obtained at the nanoscale, the amide I (1656 and 1621 cm^–1^) and II (1556 cm^–1^) vibrational modes of chitin (or its fraction in the particles) are the most dominant signals, consistent with the spectra seen in bulk ATR-FTIR spectra of the same series (Figure 2c).^32^ These chitin-specific signals decrease steadily from ChNC to 5ChsNC, where a new peak emerges at 1521 cm^–1^, which we attribute to the antisymmetric NH bending mode, δ­(NH_3_^+^), of the ammonium groups in chitosan.^33^ This is corroborated by a similar peak at 1521 cm^–1^, observed in protonated macromolecular chitosan (Figure S4).

To image the chemical heterogeneity within the nanoparticles using s-SNOM requires sensitive IR marker bands of the chitin (*N*-acetyl-d-glucosamine) and chitosan (d-glucosamine) units, which can be detected by correlating the spectral series in Figure 2b,c to the DDA in Table 1. Even though the overall intensity of the amide modes decreases in the nano-FTIR spectra as the DDA increases 10-fold to 59%, the spectral line shapes of ChNC, 1ChsNC, and 3ChsNC remain largely unchanged, i.e., very close to the chitin spectra of ChNC. At the same time, the δ­(NH_3_^+^) of chitosan remains overall undetected from ChNC to 3ChsNC due to overlap with the flank of the much more dominant amide II band at 1557 cm^–1^. Effectively, this positions the peak maximum of δ­(NH_3_^+^) at 1521 cm^–1^ very close to the isosbestic point between chitin and chitosan, and it only appears clearly at the highest DDA of 98% in 5ChsNC (Figure 2b inset). This shows that the amide modes of chitin present very sensitive IR marker bands for the degree of acetylation in the nanoparticles. Instead, the wavenumber of 1521 cm^–1^, i.e. the peak position of δ­(NH_3_^+^) of chitosan, is a quite insensitive IR marker for DDA due to residual absorption by the amide II. Therefore, the heterogeneity in DDA is more accurately derived from the amide signals, more specifically from their *absence*.

To better understand the methodology used, one can consider the heterogeneous distribution of chitin and chitosan units on the nanometer scale in the s-SNOM images of certain particles, shown in Figure 2. Note that analyses of other s-SNOM studies are based on similar considerations.^27,34,35^ Accordingly, Figure 2d shows the AFM topography of a nanoparticle along with its s-SNOM images recorded at 1556 and 1521 cm^–1^ in Figure 2e,f, respectively (2nd harmonic of the tip frequency). Due to the pseudoheterodyne detection scheme in s-SNOM,^36^ images can be further deconvolved into amplitude and phase maps that correspond to local IR reflectance and absorbance of the sample, respectively, at a given IR vibrational frequency. The s-SNOM amplitude is primarily determined by the light scattered from the sample. The corresponding amplitude maps of organic materials are, therefore, overall wavelength-independent and correspond to the AFM topography. The AFM and s-SNOM amplitude images in Figure 2d–2f are consistent with this assertion showing the same shape of the particle, that is, the highest areas of the particle coincide with the lowest amplitude (i.e., reflectance). Instead, s-SNOM phase maps in Figure 2e,f demonstrate a very different nanoscale IR absorption of the particle at both wavelengths. The phase image at 1521 cm^–1^ (Figure 2f) is consistent with the AFM and amplitude maps, with a maximum phase signal at the highest regions of the particle. However, at 1556 cm^–1^ the highest phase signal is detected at the edge of the particle and drops off toward the center. This is direct evidence for a higher density of chitin units at the edge, i.e., a higher local amide II absorbance of acetyl groups, and a patch of high DDA within the body of the particle due to a low amide II absorbance of acetyl groups. The inverted picture is not detected at 1521 cm^–1^ due to a combination of δ­(NH_3_^+^) and residual amide II absorbance, as discussed above.

Using the amplitude and phase maps at 1556 cm^–1^ as an indicator for the local DDA, we therefore imaged collections of particles along the series from ChNC to 5ChsNC (Figure 3a–f) along with their topographic images (Figure 3g,i,k). For ChNCs, we detected consistent amplitude and phase images throughout, suggesting a homogeneous distribution of acetylation with a spatial resolution of 20 nm, as shown using a representative area in Figure 3a,b. To further highlight this observation, we chose a representative particle and extracted amplitude and phase line-scan profiles (see dashed line over the indicated particle in both images, (Figure 3a,b) for a direct comparison in Figure 3h. After scaling (and inverting the amplitude), we observe that both observables provide a nearly perfect match, as the IR absorption of the amide II is directly proportional to the total mass that causes the loss in amplitude. However, for 1ChsNC to 5ChsNC (representative images in Figure 3c–f), we observe many particles with a heterogeneous distribution of DDA as described initially in Figure 2. Here, the amplitude and phase profiles (Figure 3j,l) of individual particles differ considerably—these differing regions show up to ∼150 nm-large patches within individual particles with a considerably increased DDA.

**Figure 3:**
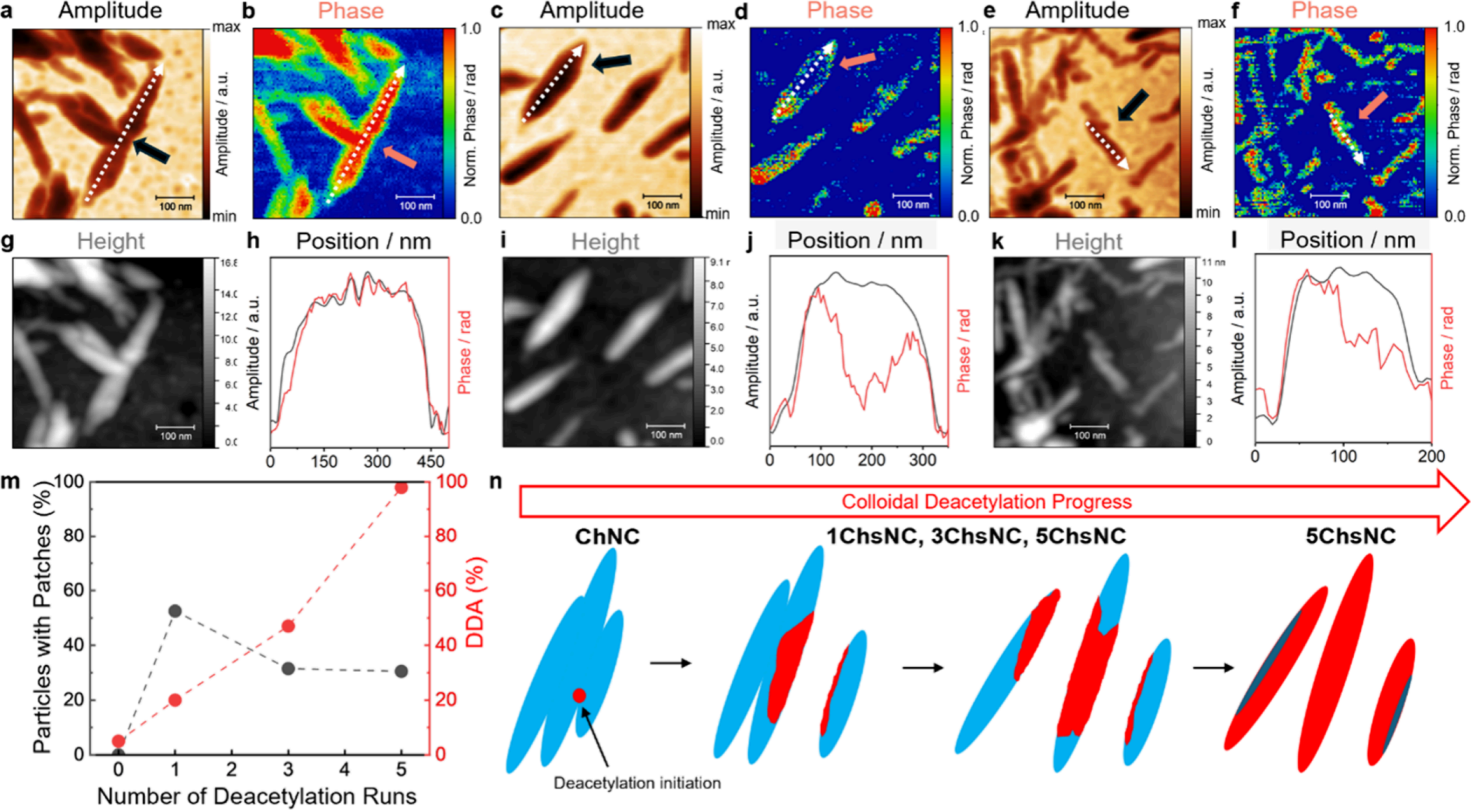
Representative s-SNOM amplitude (left) and phase (right) imaging at 1556 cm^–1^ of (a, b) ChNC, (c, d) 1ChsNC, and (e, f) 5ChsNC demonstrating nonhomogeneous patterning of chitosan during the deacetylation cycles. Topographic imaging and line scan profiles of individual nanocrystals from the white dashed arrows are shown for (g, h) ChNC (i, j) 1ChsNC and (k, l) 5ChsNC. (m) Distribution of particles with patches on the nanocrystals manually counted from the phase maps taken at 1556 cm^–1^ (*n* > 26 for each *x*ChsNC). It is to be noted that no patches can either mean that the entire rod is chitin (ChNC) or chitosan (5ChsNC). (n) Schematic representation of colloidal deacetylation, in which deacetylation preferentially occurs adjacent to already deacetylated chains, resulting in a nonhomogeneous surface deacetylation of the nanocrystals.

Using the difference between amplitude and phase maps of particles, we counted the proportion of particles with or without patches (*n* > 26) for the *x*ChsNC series, while also conducting additional mapping to confirm its abundance (Figures S5–S7). We find no patchy particles in ChNCs, and the largest number of patchy particles (53%) in 1ChsNCs, which then decreases to ∼31% after the next deacetylation steps (Figure 3m). It should be noted that the seemingly discrepant difference to the DDA in Table 1 can be explained by considering that the fraction of nonpatchy particles in the xChsNC samples could be either remaining chitin, fully deacetylated chitosan, or particles with patches that are smaller than our resolution of 20 nm.

For further quantification of the patch size found as a function of deacetylation (analysis method found in Supporting Information), we used amplitude and phase difference maps to determine the area of patches per nanoparticle average, with representative difference maps found in Figures S8–S11, and a plot summarizing the proportion of patch area for the *x*ChsNC series (Figure S12).

Overall, the visualization of these chitosan-rich patches indicates that deacetylation proceeds nonrandomly on the surface of individual chitin particles. Deacetylation is biased toward chitin chains adjacent to pre-existing chitosan units, where local disorder and swelling facilitate OH^–^ penetration and expose additional interior chains locally (Figure 3n). Although a shrinking-core model can still describe colloidal deacetylation, its underlying assumptions such as particle geometry, uniform diffusivity, and homogeneous core recession may need to be adjusted and it would be of interest for future work to adapt modified kinetic models for colloidal interfacial reactions for biopolymer particles.

### Mobile Chitosan Chains Lead to Loss of Crystallite Bundling

In parallel to the visualization of a nonheterogeneous deacetylation mechanism, a prominent decrease in crystallinity is also seen in the deacetylated nanocrystals as characterized using pXRD of the lyophilized *x*ChsNC series (Figure S13), with the greatest decrease of all α-chitin reflections in 5ChsNC. This prompted us to explore how the newly formed chitosan units arrange themselves within the rod-like framework that is visible in cryoEM (Figure 1b–e) on the nanoscale. Selected area electron diffraction (SAED) experiments were done on the nanocrystal series on dry TEM grids in order to evaluate the overall crystalline arrangement of individual nanocrystals (Figure 4a–j). For ChNC, 1ChsNC, and 3ChsNC, all nanocrystals featured α-chitin reflections, demonstrating that the objects seen under TEM are predominantly still organized in the α-chitin crystal structure. Remarkably, for 5ChsNC, which is mostly deacetylated, two populations of nanocrystals existed: some nanocrystals’ diffraction patterns displayed a disordered rod with only weak diffraction intensity observed along the fiber direction, while others displayed α-chitin reflections. Furthermore, s-SNOM imaging and dried grid TEM reveal that no solubilized chitosan oligomers are precipitated in the bulk, indicating that the glucosamine units present in the sample are attached to the nanoparticles observed in cryoEM. From this, we infer that amorphous chitosan chains that are partially solubilized still exist attached to a rigid core of either α-chitin or densely packed chitosan chains in a disordered rod-like configuration (Figure 4k).

**Figure 4:**
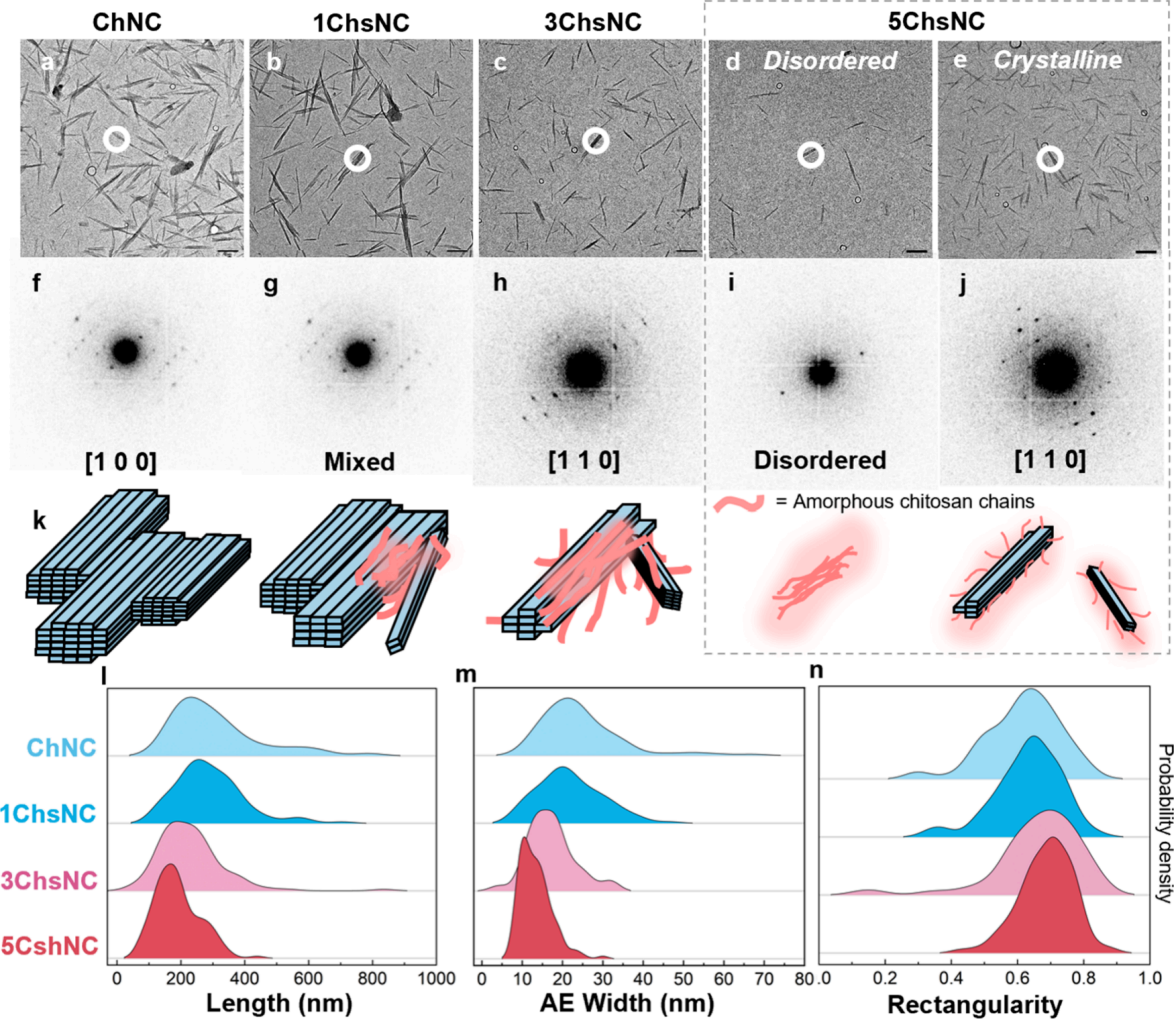
(a–e) TEM micrographs of ChNC to 5ChsNC and (f–j) their corresponding selected area electron diffraction patterns. (k) Schematic depicting the formation of amorphous chitosan chains attached to an α-chitin crystallite during the deacetylation procedure. (l) Length and (m) area-equivalent width distributions of nanocrystals of the series from dry TEM. (n) Rectangularity measurements demonstrating the decrease in bundling with increasing deacetylation. Scale bars for (a–e) are 200 nm.

To further investigate the arrangement of chitosan chains, we performed liquid-state ^1^H NMR of the colloidal samples, which demonstrate labile chains of chitosan susceptible to tumbling being visualized in the liquid-state NMR experiment (Figure S14). By using sodium 2,2-dimethyl-2-silapentane-5-sulfonate (DSS) as an internal standard, the amount of labile glucosamine units can be quantified. In this case, it can be seen that for 5ChsNC, roughly 16% of the total glucosamine units (on a mol ratio) is quantifiable using ^1^H NMR, meaning that 16% of the chitosan residues act as solubilized chains while the rest make up the rigid rod, not contributing to the NMR signal due to its rigid molecular packing and slow molecular dynamics. If chitosan oligomers were present in the 5ChsNC suspension, a significantly higher quantity of glucosamine signal would have been expected in the ^1^H NMR experiment.

To fully visualize the solubilized chitosan chains, the 5ChsNC sample was flooded with 0.1 M NaOH until pH ∼ 12, which allowed for full deprotonation of all glucosamine residues (Figure S15). Such treatment induces aggregation between the individual nanoparticles of chitosan, but also induces a “rough” surface on each individual nanocrystal, suggesting that the solubilized chitosan chains fully aggregate onto the nanorod due to charge neutralization.

We use the dried TEM images to quantify the dimensions of the nanoparticles, visualizing a decrease in the size of the nanoparticles seen under TEM on dried grids (Figure 4l,m) with increasing deacetylation. We attribute this to the combined effects of increased electrostatic repulsion and steric hindrance. ChNCs, like cellulose nanocrystals, are made up of a collection of individual crystallites “bundled” together, with varying degrees of bundling happening for each nanoparticle.^37^ This degree of bundling can be quantified using a “rectangularity” parameter, *R*, where *R* = 1 dictates an ideal rectangular profile for the measured nanoparticle, which is a strong indicator for an isolated crystallite. Manual tracings of the particles seen in dried grid TEM were performed (*n* > 100) (Figure S16). The mean rectangularity profiles of the nanocrystals (Figure 4n) increase with deacetylation, meaning that 5ChsNC primarily exists as individual nanorods, with very few or limited bundles of nanocrystals together. This debundling with increasing deacetylation has been seen in other works, with the theory that during deacetylation, electrostatic repulsion from a small population of glucosamine units in neutral pH conditions (assuming a p*K*_a_ ∼ 6.3 for chitosan) at the surface of individual chitin crystallites will drive them to ‘unstick” from the raft-like bundle configuration they are in originally.^10^ This in essence could describe the main reason for the “patchiness” found using s-SNOM, which correlates well with the debundling.

### Molecular Dynamics Simulations Explain the Presence of Patchy Surfaces on Chitin Rods

To further investigate how the chitosan chains organize on the crystalline chitin rods, we performed molecular dynamics (MD) simulations. Our main objective was to understand the reason behind the formation of the patchy surfaces at a high deacetylation degree, as demonstrated in Figure 3. A model for a partially deacetylated nanorod of ChNC was made by building a chitin crystal^25,26^ (shown in gray in Figure 5a) and then replacing the last column of the crystal with chitosan chains (shown in red). Since the deacetylation reactions were carried out in a highly basic conditions (NaOH 40 wt %) and final colloidal suspensions dialyzed in pH-neutral conditions, our model chitosan chains were not protonated. After 100 ns of equilibration in the unprotonated state, the chitosan layer begins to detach from the chitin crystal (Figure 5b). This indicates weaker interactions between chitosan chains and the chitin crystal compared to chitosan-chitosan interactions. Interestingly, when equilibration continues for 400 ns, the detached chitosan layer moves back toward the chitin crystal and remains adsorbed to it throughout the simulation (Figure 5c). The side view of the system is presented in Figure 5d and it shows that the chitosan layer keeps its periodicity along the fiber axis. This can explain the diffraction spots observed along the fiber axis for disordered structures in 5ChsNC in Figure 4i. These simulations further suggest the fact that in the partially deacetylated case (1ChsNC, 3ChsNC), the chitosan chains tend to remain in the proximity of the chitin crystal, forming an amorphous array. We further studied the behavior of the system in acidic conditions. In this case, the chitosan chains were protonated and Cl^–^ ions were added to neutralize the charges in the system (Figure S17a). After careful equilibration of Cl^–^, the system was let to equilibrate for longer. Interestingly, even 50 ns was enough to observe the dissociation of chitosan chains from the chitin crystal. This is shown in Figure S17b,c. An all-chitosan system in protonated state was also constructed to study the self-assembly of protonated chitosan chains in acidic condition (Figure S17d). Here also the protonated chitosan chains were fully dissociated (Figure S17e,f) which is consistent with recent findings.^200^

**Figure 5:**
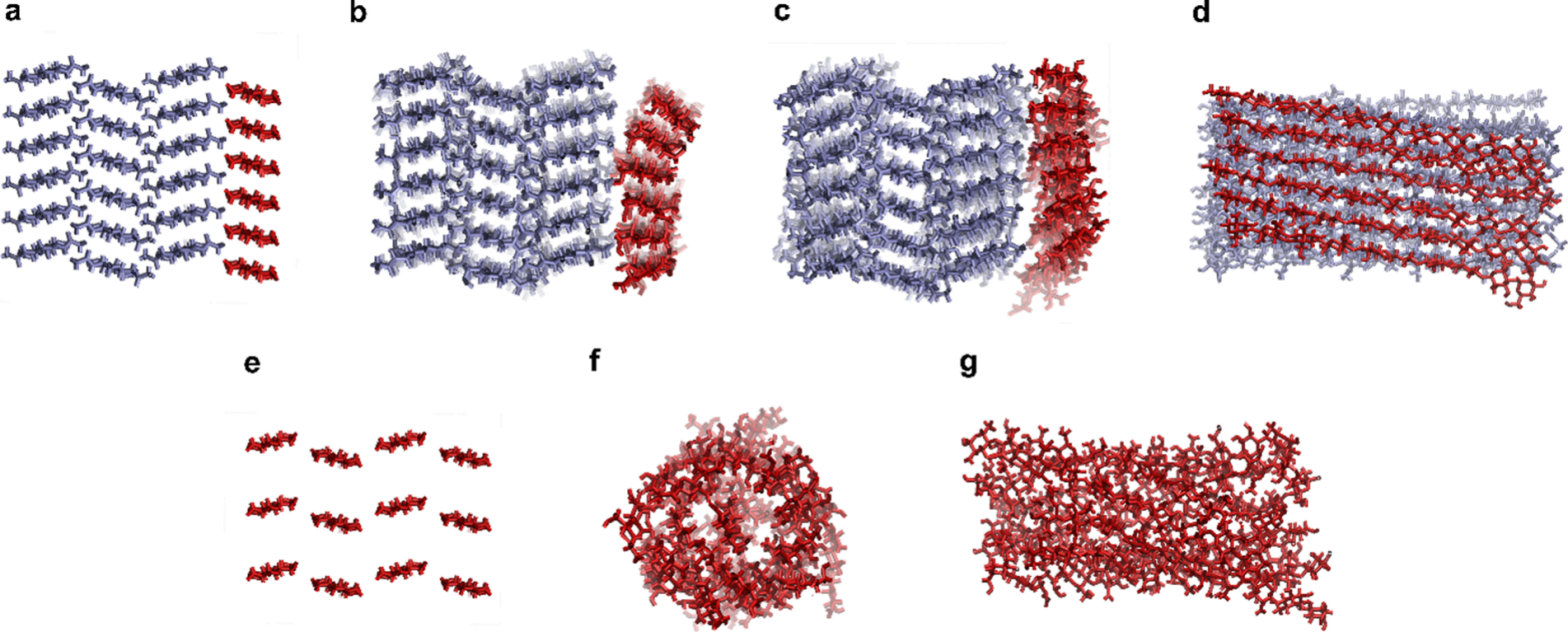
Molecular dynamics (MD) simulations depicting (a) a model chitin crystal, where all the chains on the edge of the crystal, shown in red, are completely deacetylated (turned to chitosan). (b) The model system after equilibration in the unprotonated state in water for 100 ns. The chitosan layer starts to come off the chitin crystal. The water molecules are not shown here for representation purposes. (c) The model system after a 400 ns simulation in the unprotonated state in water. The chitosan layer has lost the crystalline organization but stays adsorbed to the crystal. (d) Side-on view of a chitosan layer on top of a chitin crystal in Figure 5c. (e) A model system of only chitosan chains before equilibration. (f) The all-chitosan system after 100 ns simulation in the unprotonated state in water and (g) side-on view of chitosan chains in Figure 5f.

We then carried out simulations under highly basic conditions, as in the deacetylation reaction, to examine the distribution of the alkali species (OH^–^) around the chitosan patch in comparison to the pure chitin surface. Figure S18a–d shows that the concentration of OH^–^ ions increased around the fully deacetylated chitosan patch compared with the chitin patch. The increased OH^–^ concentration around the chitosan patch supports our hypothesis that deacetylation preferentially happens near already deacetylated chains. Expectedly, the number of hydrogen bonds between chitosan chains and surrounding OH^–^ was also two times higher than between chitin and OH^–^.

To study how fully deacetylated chitosan chains self-assemble in water in a nonprotonated state, 12 chitosan chains were placed next to each other to make an all-chitosan system (Figure 5e). Interestingly, after 100 ns of equilibration, the chitosan chains pack tightly together with a twist. Figure 5f,g show a cross-section and side view of such system after equilibration. These simulations suggest that, in the fully deacetylated case (5ChsNC), densely packed chitosan rods can maintain stability under neutral conditions, as observed in TEM (Figure 4i).

## Conclusions

In this work, we rationalize the colloidal deacetylation mechanism of ChNCs using a comprehensive suite of nanoscopic characterization techniques. Our results reveal that partially deacetylated ChNCs (20–60% DDA) exhibit spatially discrete glucosamine-rich patches, providing direct evidence against the classical shrinking core model, which assumes a chitin core surrounded by a progressively expanding chitosan shell. Our results suggest that the deacetylation process generates localized patchy surface domains, consistent with emerging observations in bulk chitin systems and with implications for biological functionality. Finally, we demonstrate that highly deacetylated ChsNCs (DDA > 90%) can exist as nanorods composed of partially solubilized chitosan chains organized around a dense core of either chitosan-rich domains or residual chitin crystallites. This hybrid structure challenges conventional assumptions about uniform chemical conversion in nanopolysaccharides, introducing a more nuanced model that involves intermediate, metastable architectures. Together, these findings raise fundamental questions about the mechanisms and spatial dynamics of surface functionalization in polysaccharide nanomaterials. They emphasize the importance of considering nanoscale heterogeneity during chemical modification processes, particularly for applications where surface functionality has a significant influence on performance. Anisotropic surface functionalization may be of research interest toward a variety of fields including asymmetric catalysis, biosensing, and surfactant-based applications. Finally, the multitechnique methodology presented here—including high-resolution nano-FTIR, s-SNOM, cryo-EM, and molecular modeling—offers a broadly applicable framework for probing and rationalizing the structural evolution of chemically modified cellulose and chitin nanomaterials.

## Methods/Experimental Section

### Materials

Shrimp shell chitin and potassium hydroxide were obtained from Sigma-Aldrich. Sodium borohydride and deuterium oxide were obtained from Thermo Scientific, sodium hydroxide pellets were obtained from AnalytiChem. Sodium trimethylsilylpropanesulfonate (DSS) was obtained from TCI. Hydrochloric acid (37%) was obtained from Roth.

### Purification of Chitin

Shrimp shell chitin was treated with 500 mL HCl solution (0.01 M) at room temperature for 18 h, before being neutralized with dilute NaOH solution. Base treatment was then performed with 500 mL KOH (0.5 M) at 70 °C for 3 h to remove proteins. Subsequently, 25 mL of hydrogen peroxide solution (30% w/v) was added three times with 1-h intervals. Afterward, the reaction was quenched with cold water and vacuum filtered with an acetone wash. The final yield was 11.7 g (58.5%).

### Acid Hydrolysis of Chitin to Form ChNCs

12.4 g of purified chitin was subjected to acid hydrolysis using 350 mL of HCl solution (3 M) at 105 °C using the EasyMax reactor at 300 rpm for 4.5 h. After the reaction, the vessel was quenched with excess cold water and then centrifuged three times at 8000 rpm for 10 min. The pellets were then resuspended in Milli-Q water and dialyzed against Milli-Q water until the conductivity of the dialysis bath remained constant between days. After dialysis, 1 M HCl solution was added until the pH of the suspension was ∼4. Ultrasonication was then performed on the ChNC suspension using a tip sonicator at 30% amplitude for 4.5 min, with a pulsing time of 10 s on and 15 s off. After sonication, the suspensions were filtered through cellulose membranes with pore channels of 8 μm, then 0.8 μm. Roughly 700 mL ChNC suspension with a concentration of 0.6% (w/w) was obtained. The final yield was 4.27 g (34%). Note that the remaining ChNC suspension kept from the deacetylation procedure was dialyzed against milli-Q water, and this sample will be known as **ChNC**. More information is available in the Supporting Information File.

### Colloidal Deacetylation of ChNCs to Produce ChsNCs

600 mL of 0.6 wt %, **ChNC** suspension (3.66 g) was centrifuged with the addition of 0.1 M aqueous NaOH until basic (pH ∼ 10). The pellets were then redispersed in a total of 183 g of 40% (w/w) aqueous NaOH solution in a 500 mL round-bottom flask (Scheme S1a). Immediately after redispersion, 0.183 g of NaBH_4_ was added to the reaction mixture, resulting in a total ratio of 20:1:1000 ChNC to NaBH_4_ to 40% NaOH solution. The reaction mixture was then heated to reflux (105 °C) for 18 h. The reaction mixture was subsequently quenched with cold water before being centrifuged until pH neutral at 8000 rpm for 10 min and 8 °C. More information is available in the Supporting Information File.

## Supplementary Material



## Notes

Additional information on the in-depth methodology of the synthesis of ChNCs, colloidal deacetylation to achieve ChsNCs, materials characterizations (conductometric titration, electron microscopy, DLS, XRD, FTIR, NMR, s-SNOM), analysis details for MD simulations, and s-SNOM quantification (DOCX)
