# Supplementary material for: Colloidal Deacetylation of Chitin Nanocrystals Results in Amorphous and Patchy Chitosan Chains
Source: ACS Nano. 2026 Apr 10;20(16):12347–56. doi: 10.1021/acsnano.5c21467 (PMC13131042; doi:10.1021/acsnano.5c21467)
Supplement: Supplementary file 1 [file nn5c21467_si_001.docx]

**Supplemental Information**

*Colloidal Deacetylation of Chitin Nanocrystals Results in Amorphous and Patchy Chitosan Chains*

Tony Jin^a^, Saskia Heermant^b^, Hanieh Mianehrow^a^, Thomas G. Parton^a^, Antonio Carone^a^, Ruslan Nedielkov^c^, Yu Ogawa^a,d^, Jacek Kozuch^b,e*^, Silvia Vignolini^a*^

^a^ Department of Sustainable and Bio-inspired Materials, Max Planck Institute of Colloids and Interfaces, Am Muhlenberg 1, 14476 Potsdam, Germany

^b^ Department of Physics, Freie Universität Berlin, Arnimallee 14, 14195 Berlin, Germany

^c^ Department of Chemistry, University of Potsdam, Karl-Liebknecht Straße 24-25, 14476 Potsdam, Germany

^d^ Univ. Grenoble Alpes, CNRS, CERMAV, 38000 Grenoble, France

^e^ Institute of Physical Chemistry and Theoretical Chemistry, BRICS – Braunschweig Integrated Centre of Systems Biology, Rebenring 56, 38106 Braunschweig, Germany

^*^*Corresponding author*

**Materials**

Shrimp shell chitin and potassium hydroxide were obtained from Sigma Aldrich. Sodium borohydride and deuterium oxide were obtained from Thermo Scientific, sodium hydroxide pellets were obtained from AnalytiChem. Sodium trimethylsilylpropanesulfonate (DSS) was obtained from TCI. Hydrochloric acid (37 %) was obtained from Roth.

**Methods**

*Purification of chitin*

A modified protocol from a previous publication is used.^1^ Shrimp shell chitin was treated with 500 mL HCl solution (0.01 M) at room temperature for 18 h, before being neutralized with dilute NaOH solution. Base treatment was then performed with 500 mL KOH (0.5 M) at 70 °C for 3 h to remove proteins. Subsequently, 25 mL of hydrogen peroxide solution (30 % w/v) was added three times with 1-hour intervals. Afterwards, the reaction was quenched with cold water and vacuum filtered with an acetone wash. The final yield was 11.7 g (58.5 %).

*Acid hydrolysis procedure to form chitin nanocrystals (ChNCs)*

A modified protocol from a previous publication was used.^1^ 12.4 g of purified chitin was subjected to acid hydrolysis using 350 mL of HCl solution (3 M) at 105 °C using the EasyMax reactor at 300 rpm for 4.5 h. After the reaction, the vessel was quenched with excess cold water and then centrifuged three times at 8000 rpm for 10 minutes. The pellets were then resuspended in Milli-Q water and dialyzed against Milli-Q water until the conductivity of the dialysis bath remained constant between days. After dialysis, 1 M HCl solution was added until the pH of the suspension was ~ 4. Ultrasonication was then performed on the ChNC suspension using a tip sonicator at 30% amplitude for 4.5 minutes, with a pulsing time of 10 s on and 15 s off. After sonication, the suspensions were filtered through cellulose membranes with pore channels of 8 μm, then 0.8 μm. Roughly 700 mL ChNC suspension with a concentration of 0.6 % (w/w) was obtained. The final yield was 4.27 g (34 %). Note that the remaining ChNC suspension kept from the deacetylation procedure was dialyzed against milli-Q water, and this sample will be known as **ChNC**.

*Colloidal deacetylation procedure to create deacetylated nanocrystals (ChsNCs)*

A modified protocol from a previous publication was used.^2^ 600 mL of 0.6 wt%, **ChNC** suspension (3.66 g) was centrifuged with the addition of 0.1 M aqueous NaOH until basic (pH ~ 10). The pellets were then redispersed in a total of 183 g of 40 % (w/w) aqueous NaOH solution in a 500 mL round-bottom flask (Supplemental Scheme S1a). Immediately after redispersion, 0.183 g of NaBH_4_ was added to the reaction mixture, resulting in a total ratio of 20:1:1000 ChNC to NaBH_4_ to 40% NaOH solution. The reaction mixture was then heated to reflux (105 °C) for 18 h. The reaction mixture was subsequently quenched with cold water before being centrifuged until pH neutral at 8000 rpm for 10 minutes and 8 °C. Afterward, the total mass of wet pellets was separated: one-third portion was redispersed in milli-Q water and added HCl (0.1 M) until pH 5 (Supplemental Scheme S1b). This was then sonicated at 4.5 minutes using a pulsing time of 10 s on and 15 s off at 30 % amplitude, and dialyzed against milli-Q water. This sample is known as **1ChsNC**, with a total yield of 53 % (0.64g) with respect to one-third of the starting ChNC (3.66 g).


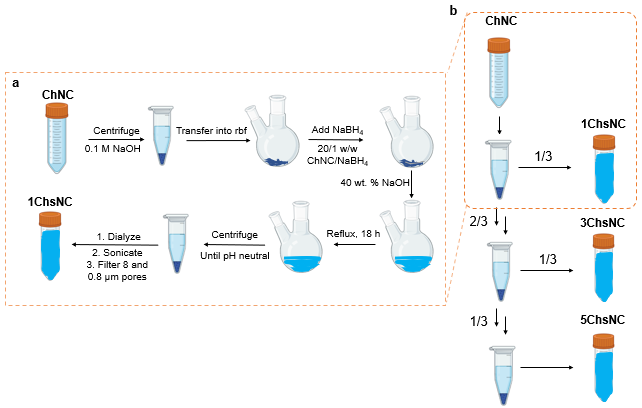


**Supplemental Scheme S1**: Schematic demonstrating (a) the first deacetylation procedure and the pathway towards 3ChsNC and 5ChsNC from the same batch of ChNCs.

The remaining two-thirds portion was subjected to the next round of deacetylation, following the protocol above with a mass ratio of 20:1000 of nanocrystals to 40 % NaOH solution. This was repeated for a third time, in which the pellets were split in half, with one being subjected to post-treatment sonication and dialysis against milli-Q water to give **3ChsNC** with a yield of 0.60 g (49 % ) with respect to one-third of the starting ChNC (3.66 g).

The last third was subjected to two more rounds of deacetylation following the protocol above, with a mass ratio of 20:1000 of nanocrystals to 40 % NaOH solution. Finally, after 5 deacetylation rounds, the remaining pellets after centrifuge cleaning were subjected to sonication in acidic conditions, and dialyzed against milli-Q water to give **5ChsNC**, with a yield of 0.62 g (51 %) with respect to one-third of the starting ChNC (3.66 g).

*Calculation of theoretical maximum surface amine content*

The crystal size, as determined by cryoEM, for a single ChNC is 12.6 nm in width and 168 nm in length. This corresponds to 52 chains accessible on the surface of the chitin crystal (Figure 1f), each with approximately 170 acetyl groups pointing outward and prone to deacetylation (this calculation is based on a smaller model of the chitin crystals generated using GROMACS software). In total, there are roughly 8800 accessible acetyl groups on the surface of a real chitin crystal. This, divided by the mass of a single ChNC as determined by cryoEM, calculated by using a cylindrical volume of a single nanocrystal and a density of 1.425 g cm^-3,^ yields a theoretical maximum surface amine content of 480 mmol kg^-1^.

*Electron Microscopy*

Transmission electron microscopy and electron diffraction measurements were performed using a JEOL JEM 2100Plus (Jeol, Japan), operated at 200 kV, equipped with a Gatan RIO 16 camera (Gatan Inc., U.S.A.) and a MerlinEM hybrid pixel detector (Quantum Detectors, U.K.). Cryo-frozen samples were prepared using an EM GP2 Automatic Plunge Freezer (Leica microsystem, Germany). Selected area electron diffraction (SAED) experiments were performed with an aperture that selected an area with a diameter of 200 nm. Nanobeam electron diffraction (NBED) was performed with a focused electron probe with a diameter of 80-100 nm.

*DLS*

Dynamic light scattering and zeta potential measurements of the ChsNC series were measured from dilute suspensions of the ChsNC series (~0.005 wt% ) in disposable cuvettes and measured using a Malvern Zetasizer in triplicate.

*^13^C MAS NMR*

Solid-state NMR measurements were carried out on a Bruker NEO spectrometer operating at 400.13 MHz, equipped with a magic-angle spinning (MAS) PH MAS 400 S1 BI4 N-P/H VTN double resonance probe using 4 mm rotors (Bruker BioSpin, Rheinstetten, Germany). All spectra were acquired and processed using Bruker TopSpin 4.4 software. The measurement temperature was kept at 298 K. The MAS rate throughout the experiments was at 10 kHz. The samples of chitin and chitosan nanocrystals were ground in a porcelain mortar and filled into 4 mm ZrO_2_ rotors with Kel-F® drive caps. ^13^C spectra of chitin and chitosans were acquired using a standard cross-polarization (CP) pulse sequence with 2.5 µs ^1^H 90° hard pulse and 2 ms contact pulse. Each ^13^C spectrum was composed of 512 scans with an acquisition time of 34 ms and a recycling delay of 5 s. The spectra were externally referenced to the carbonyl signal of the α-glycine (175.7 ppm).

*^1^H NMR*

A known amount of lyophilized nanocrystal powder (around 6-10 mg) was resuspended in roughly 1 mL of D_2_O using bath sonication and vertexing before a known amount of DSS was added (6 – 11 mg). Proton NMR experiments were carried out on a Bruker Avance 400 spectrometer for 16 scans.

*Powder XRD*

X-ray diffraction spectra were acquired using a Rigaku Smartlab X-ray diffractometer equipped with a PhotonMax X-ray source, scanned with a 2θ range between 5 – 40° with an increment of 0.01°.

*Conductometric Titration*

To a 100 mL beaker was charged 50 mg of nanocrystals (in ~ 1 wt % suspension form), 100 mL of milli-Q water, 3 mL of 0.1 M HC, and 0.5 mL of 0.01 M NaCl. Subsequently, an automatic titrator (Metrohm Dosino 800) was used to titrate a 0.097 M solution of NaOH (exact molarity calculated by titration with a standardized 0.1 M HCl solution) in 0.01 mL increments.

*ATR-FTIR*

Spectra were collected from 4000-500 cm^-1^ for 32 scans in absorbance mode using a Thermo Scientific Nicolet iS5 in ATR mode.

*Scattering-type Scanning Near-field Optical Microscopy (s-SNOM)*

Samples containing the nanocrystals were deposited on a flat template-stripped gold^3^ and dried for s-SNOM and nanoFTIR measurements using a commercial microscope (neaSNOM, attocube systems AG, Germany). The system is equipped with quantum cascade lasers (MIRCat, Daylight Solutions) for s-SNOM measurements and an IR-Fiber-Laser for nanoFTIR measurements. For both methods, commercial AFM tips with a tip apex diameter of 30 nm (Arrow NCPt, NanoWorld, Switzerland) were used at resonant frequencies around 280 kHz in tapping mode and oscillation amplitudes set to 60 nm. To obtain nanoFTIR spectra, background spectra were taken on pure gold spots and subtracted from spectra measured on different spots on multiple nanocrystals (N=10 for ChNC, 1ChsNC, and 3ChsNC, N=30 for 5ChsNC due to lower signal-to-noise ratios in those spectra). Each spectrum consists of 100 averages, each with an integration time of 6 ms. All spectra were averaged and finally baselined. Errors indicated in the spectra (Fig. 2b) result from the standard deviations among the individual spectra. During s-SNOM recordings, the AFM topography, s-SNOM amplitude and phase images (demodulated at higher harmonics of the tip driving frequency – data shown herein were the detected at the 2^nd^ harmonic) were recorded at random areas with the quantum cascade lasers tuned to 1557 cm^-1^, a maximum laser power of 0.7 mW, and an integration time per pixel of 9.8 ms. The same areas were measured again at 1521 cm^-1^. Images are shown with a pixel size of 10 ˟ 10 nm. For visualization of the sSNOM images, Gwyddion (v. 2.63) was used.

*Molecular Dynamics Simulations*

A finite chitin crystal model with 24 chitin chains, each with 10 N-acetylglucosamine units, was constructed using the Chitin Builder module in VMD 1.9.3.^4^ The last column of the crystal was then manually modified to create chitosan chains by removing the acetyl groups. The topology files for chitin and chitosan chains were generated using LEap program in AMBER 20 package^5^ and with GLYCAM06 forcefield.^6^ In another set of simulations, an all-chitosan system was created by placing 12 chitosan chains, each consisting of 10 D-glucosamine units, adjacent to one another. The geometry file was further modified to make a model system for protonated chitosan by manually adding Hydrogen atoms to the amine groups. All the model systems were then placed in a simulation box of 8×8×8 nm and then solvated in water using SPC/E water model.^7^ MD simulations were then performed using GROMACS 2022.^8^ The cut-off distance of 1.2 nm was used for non-bonded interactions, and long-range electrostatic interactions were modeled by PME. The simulations were performed at 298 K using V-rescale thermostat^9^ and the pressure was kept at 1 atm using C-rescale barostat.^10^ The systems were first energy minimized for 1000 steps and then equilibrated. In the case of protonated chitosan, Cl^-^ ions were added to the system to neutralize the positive charges on chitosan. Positional restraints were initially placed on the chitin and chitosan chains, so that the chains were frozen but the solvent atoms and Cl^-^ ions were free to move. The equilibration ran for 20 ns to let the Cl^-^ ions migrate near the NH3^+^ sites, then the positional restrains were released and the simulations ran for another 100 ns.

In another set of simulations, Na^+^ and OH^-^ ions were added to the simulation box until the concentration of 40 w/w% was reached. We also made another model system, in which only two chains of the chitin crystal were substituted with chitosan chains to mimic a deacetylated patch. Both systems were then equilibrated for 200 ns and the radial distribution function for OH^-^ around the chitosan and chitin patches was calculated. In this case, the temperature was set to 380 K, similar to the experimental conditions, to enhance the dynamics. The non-bonded parameters for all the ions were taken from (J. Chem. Phys. 144, 104503 (2016)).

**Additional Analysis and Discussion**

*Determination of the Relative Patch Area per Particle*

In order to estimate the relative patch area per particle for ChNC, 1ChsNC, 3ChsNC and 5ChsNC, we analyzed amplitude and phase images of individual particles according to the comparison of line scans in Figure 3 in the main text. That is, we utilized the observation that non-patchy particles show a similar response in sSNOM amplitude and phase, whereas patchy particles display distinctly different amplitude and images. The following describes the analysis in a step-by-step fashion:

1. Phase images of individual particles were corrected for slopes in the baseline, such that the surrounding of a particle was flat at a phase of zero. Amplitude images were inverted and normalized such that the surrounding was zero and the strongest response due to the particle was at 1.
2. To obtain a similar picture as in Figure 3h, j and i, the region with the highest phase signal (for instance with a threshold > 0.9) was selected and the amplitude image was scaled to the same area. Note the accuracy of this step can be affected considerably by the noise in phase images. Therefore, a smoothing of 5x5 pixels was performed and a set of thresholds of 0.7, 0.8, 0.9 and 0.95 was applied. The images of six particles of each deacylation step after this simultaneous scaling are shown in Supplemental Figures X – Y (left and middle).
3. Finally, “amplitude minus phase” images were created, where patchy regions occur with positive signal (higher response in amplitude, lower response in phase), and a noise gate was applied by setting all difference intensities < 0.1 to zero. The results are shown in Supplemental Figures X - Y (right) of each panel.

ChNC does not show any considerable difference in intensity as amplitude and phase images are highly similar. Instead 1ChsNC, 3ChsNC and 5ChsNC show patches with considerable differences, typically located at regions where inhomogeneous signal is observed already in the phase images.

1. To quantify the patch area, the number of pixels with non-zero difference intensity was divided by the number of pixels of the entire particles observed in the amplitude image (the latter is unaffected by patchiness).

The distributions of the relative patch area per particle (Supplemental Figure X) shows that the chosen approach determined a negligible patchiness of (2 ± 2)% for ChNC, while deacylation products show significant patch sizes. As for 1ChsNC, 3ChsNC and 5ChsNC, particles with a patchiness of <5% were discarded from the following statistical analysis, since one could assign them to a fraction of non-patchy particles within the samples. For 1ChsNC a main fraction shows patch areas of (19 ± 9)%, while a minor one displays a much higher area of (63 ± 7)%. The higher one disappears in the following deacylation steps and only the major fraction with (17 ± 8)% and (23 ± 13)% persists. The areas of the major fraction are similar within error margin. The increased oscillation in the distribution of 5ChsNC shows along with the larger standard deviation of the relative areas can be attributed to the noisier phase images due to the lower chitin content.

We want to note that these percentual areas do not represent directly the chitin/chitosan content, but their heterogeneous distribution. As shown by the degree of deacetylation in the main text, the overall chitin content is lowered by each deacetylation step.

**Supplemental Figures**


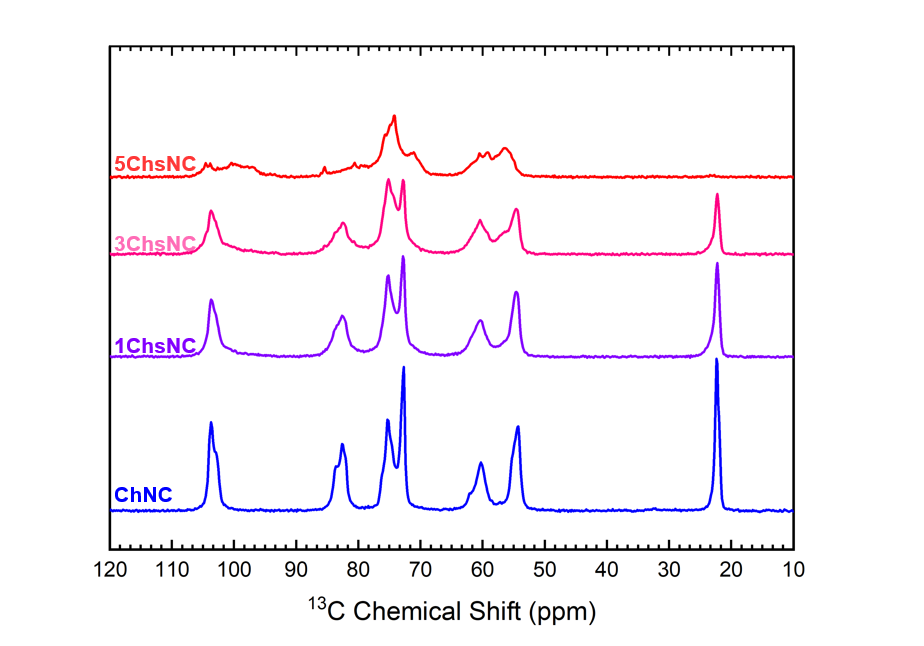


**Supplemental Figure S1**: ^13^C CP-MAS NMR of the nanocrystal series.


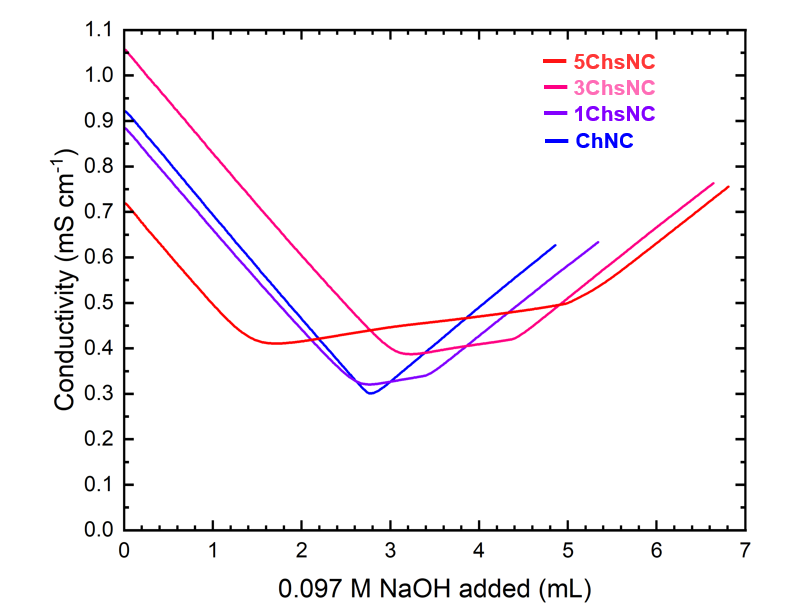


**Supplemental Figure S2:** Raw conductometric titration data of the deacetylation series. To calculate the surface amine content, three linear regression lines were plotted from each of the three regions of the titration: 1) strong acid – strong base neutralization, 2) weak acid (amine) – strong base neutralization, and 3) excess base. From these linear regression lines, the two intersection points (1-2, 2-3) and the difference between these two is the total amount of hydroxide ions, which also correlates with the total surface amine content of the suspension per weight of total nanocrystals in the suspension. Conversion to DDA as seen in the last row of Table 1 was done using the molecular weight values of N-acetyl-D-glucosamine (203.19 g mol^-1^) and D-glucosamine (179.17 g mol^-1^) for ChNC and 5ChsNC. For 1ChsNC and 3ChsNC, an average molecular weight value using the DDA ratio was utilized, which resulted in molecular weight values of 198.34 g mol^-1^ and 191.90 g mol^-1^, respectively.


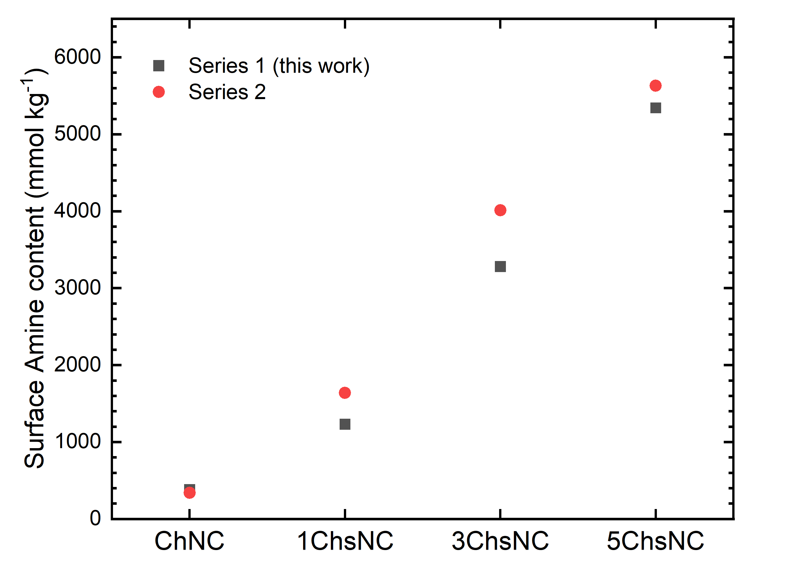


**Supplemental Figure S3:** Comparison of the series used in this work with another batch of ChsNC series, demonstrating the reproducibility of the sequential deacetylation procedure.


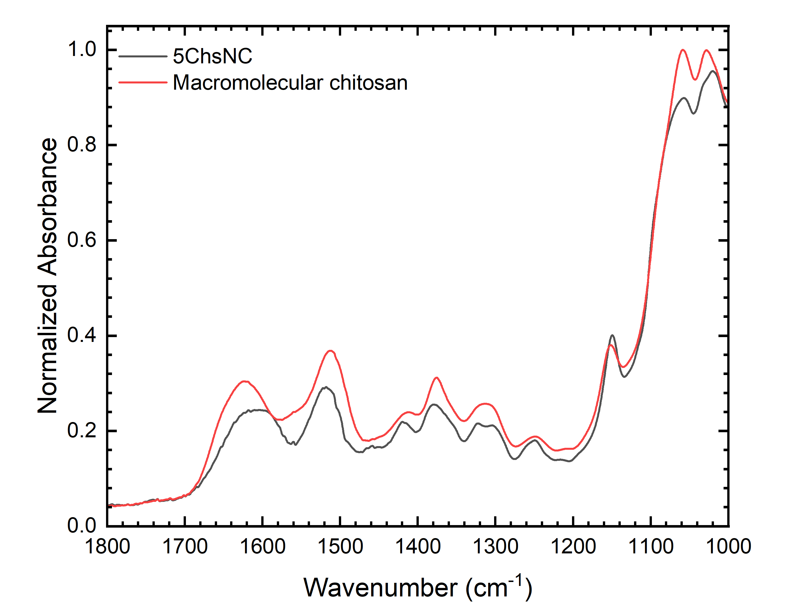


**Supplemental Figure S4:** FTIR spectra comparison between 5ChsNC and macromolecular chitosan (as-received from Sigma Aldrich) dissolved in 0.1 M HCl and then dried into a powder. The peak around 1520 cm^-1^ is associated with the N-H bending vibration of protonated -NH_3_^+^ from glucosamine.


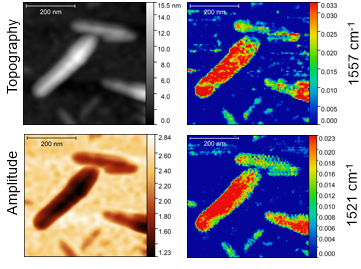


**Supplemental Figure S5:** Additional representative 1ChsNC mappings of topography, amplitude, and phase signal at 1557 and 1521 cm^-1^


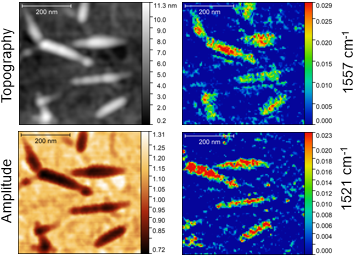


**Supplemental Figure S6:** Representative 3ChsNC mappings of topography, amplitude, and phase signal at 1557 and 1521 cm^-1^ showing “patchy” deacetylation.


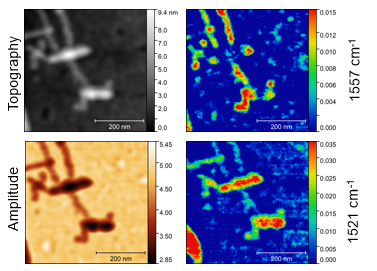


**Supplemental Figure S7:** Representative 5ChsNC mappings of topography, amplitude, and phase signal at 1557 and 1521 cm^-1^ showing “patchy” deacetylation.


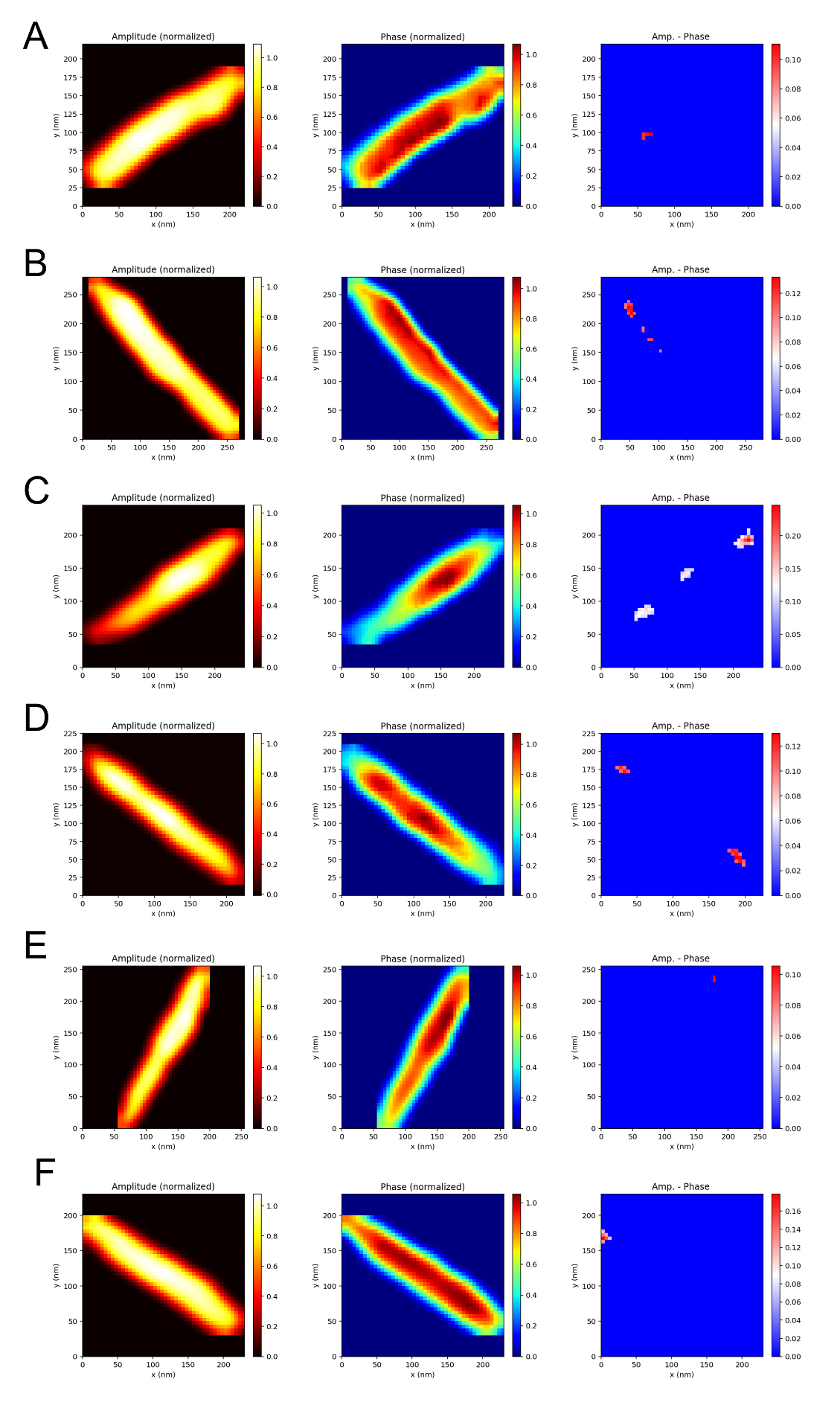


**Supplemental Figure S8**: (a-f) Inverted amplitude images (left) and phase images (middle) of ChNC particles after simultaneous scaling, as described in the SI text. The resulting difference (amplitude - phase) image is shown on the right. Cuts observed in the images originated from a mask that was used to select the particles.


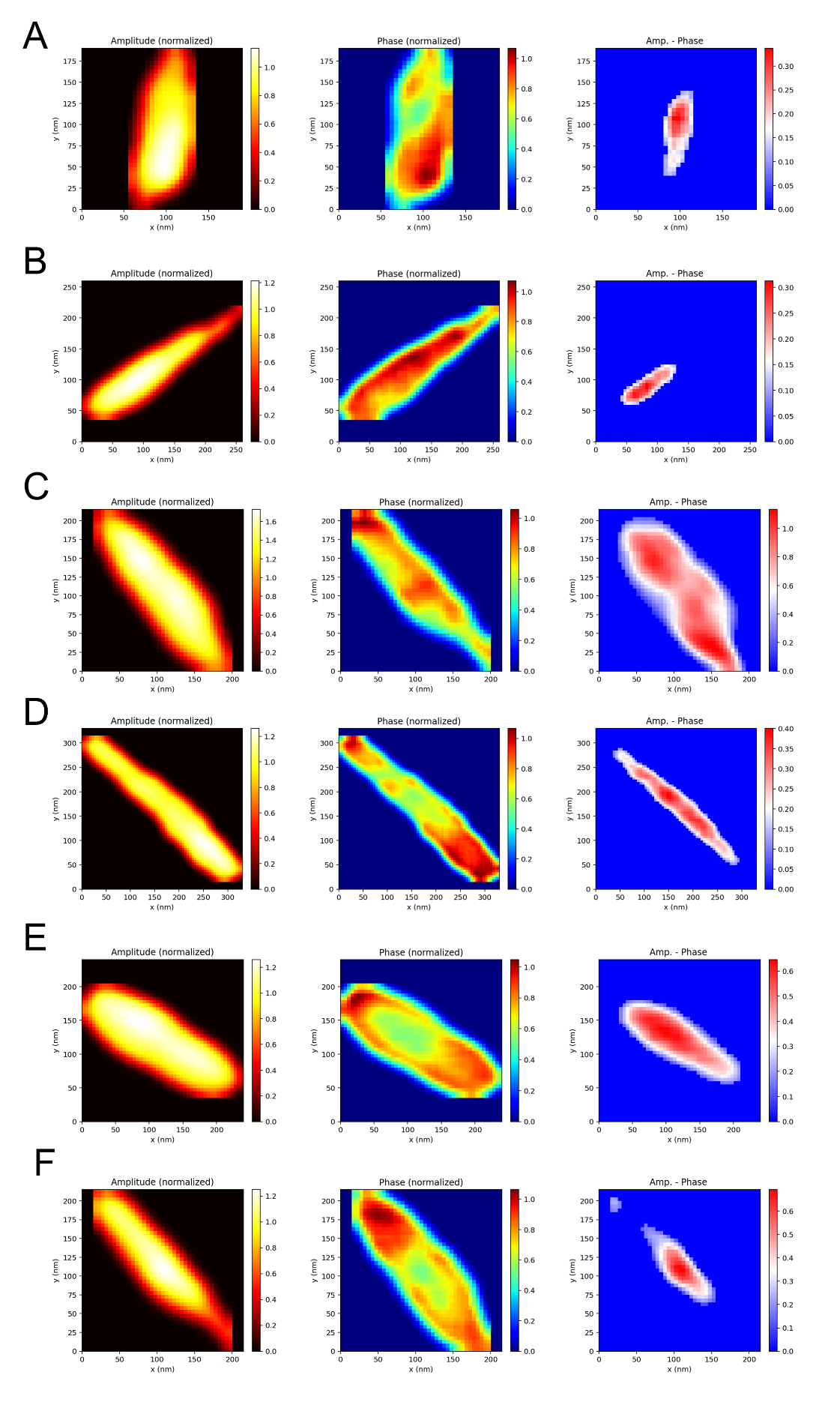


**Supplemental Figure S9:** (a-f) Inverted amplitude images (left) and phase images (middle) of 1ChsNC particles after simultaneous scaling, as described in the SI text. The resulting difference (amplitude - phase) image is shown on the right. Cuts observed in the images originated from a mask that was used to select the particles.


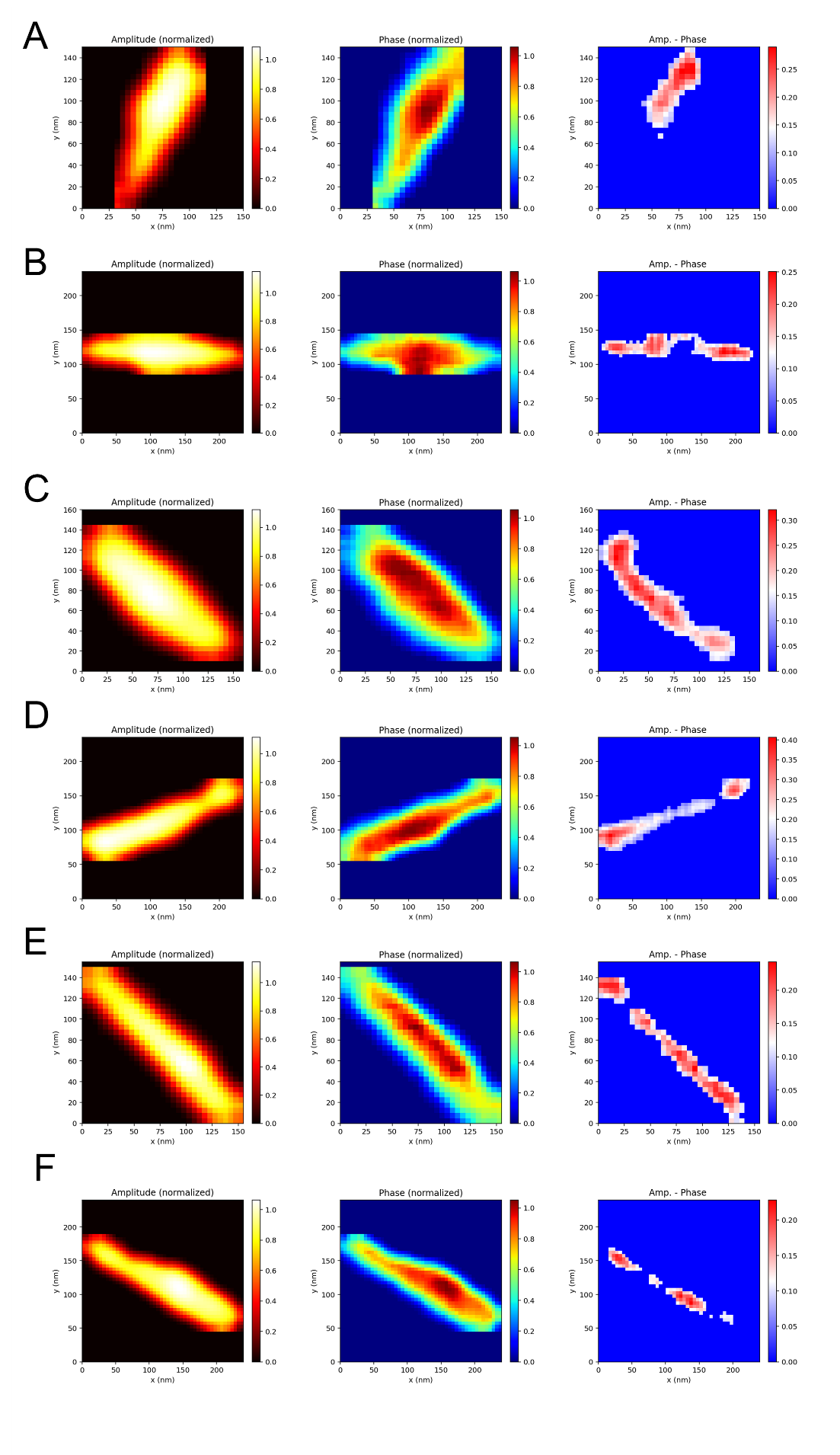


**Supplemental Figure S10:** (a-f) Inverted amplitude images (left) and phase images (middle) of 3ChsNC particles after simultaneous scaling, as described in the SI text. The resulting difference (amplitude - phase) image is shown on the right. Cuts observed in the images originated from a mask that was used to select the particles.


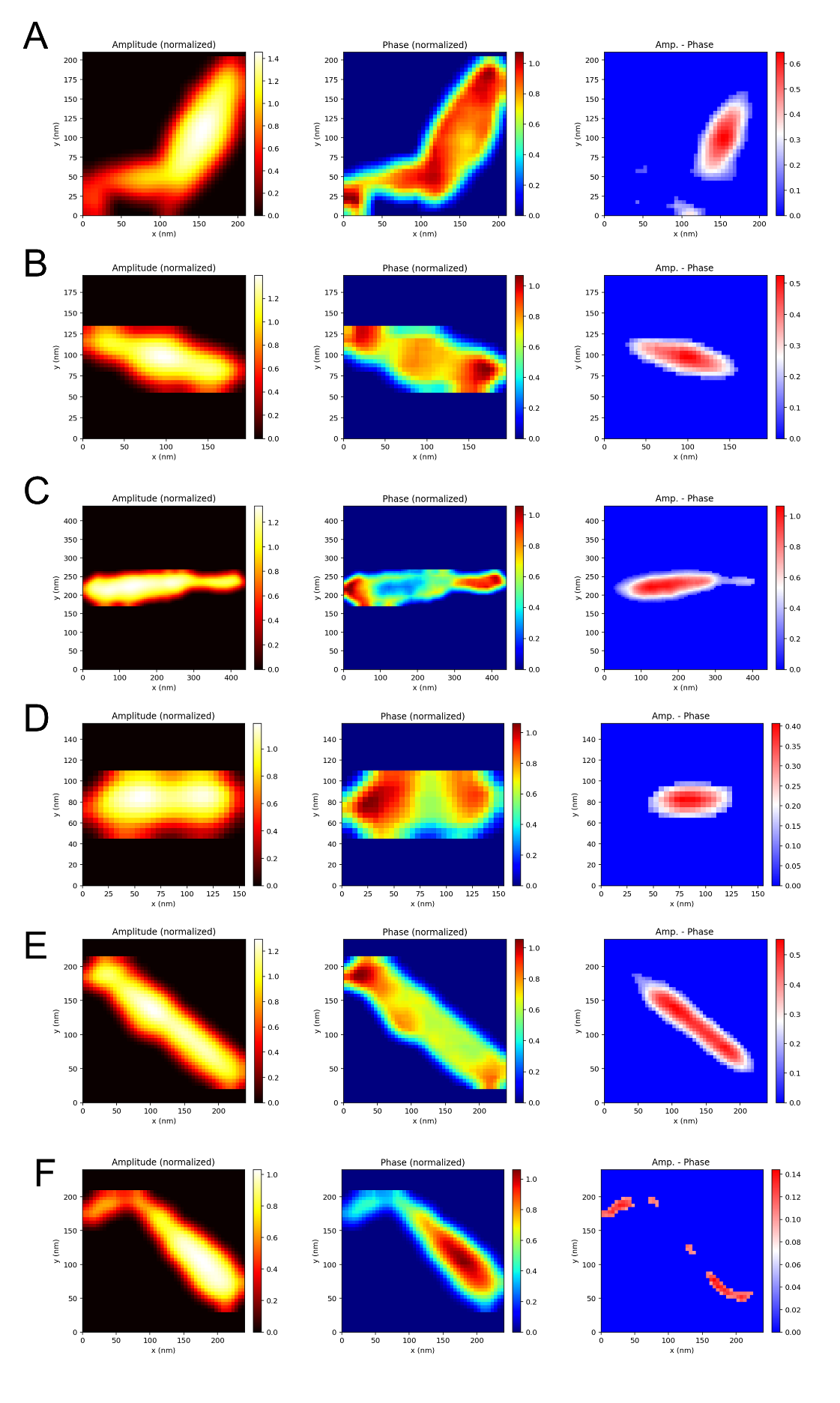


**Supplemental Figure S11:** (a-ef) Inverted amplitude images (left) and phase images (middle) of 5ChsNC particles after simultaneous scaling, as described in the SI text. The resulting difference (amplitude - phase) image is shown on the right. Cuts observed in the images originated from a mask that was used to select the particles.


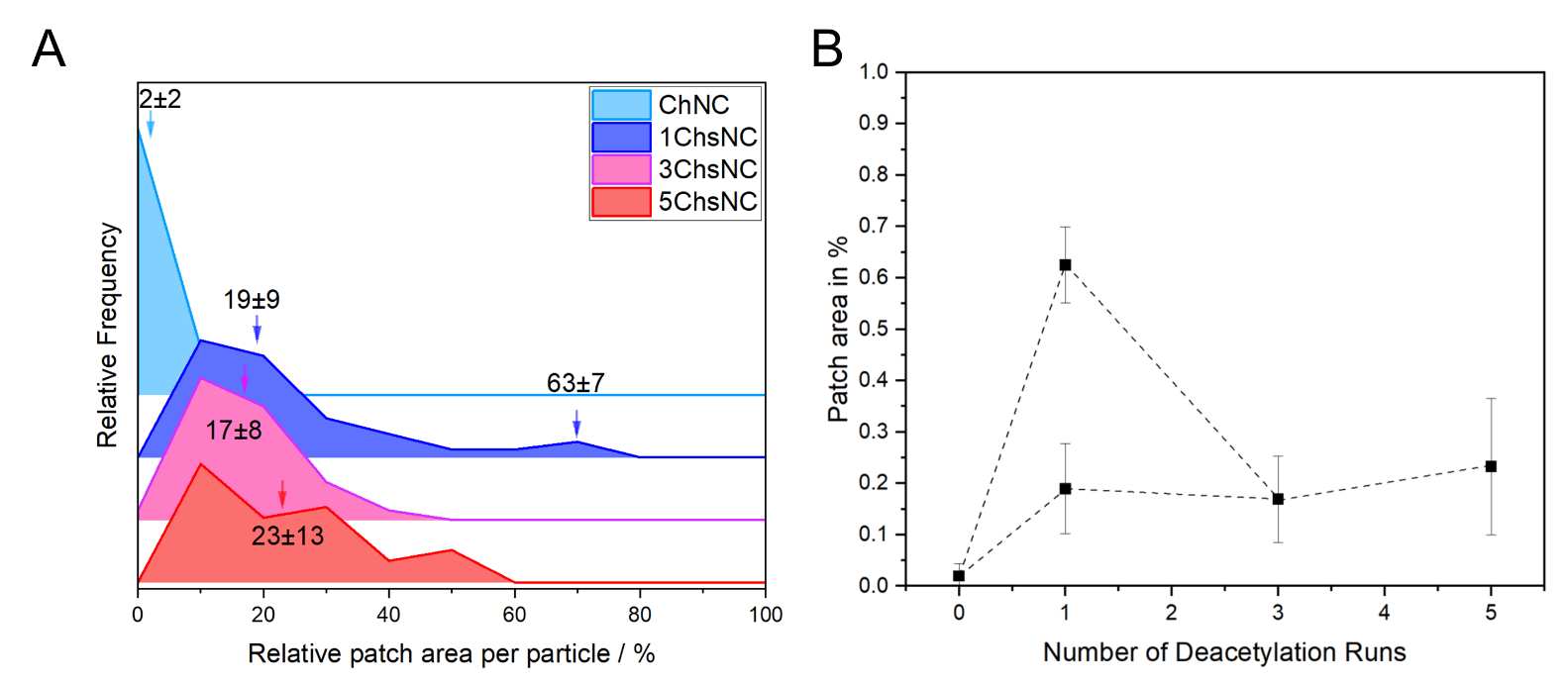


**Supplemental Figure S12:** (a) Distributions of relative patch area per particle for ChNC, 1ChsNC, 3ChsNC and 5ChsNC (colors are shown in the legend). Averages and standard deviations are indicated in the graph with arrows of the same colors. The average for 1ChsNC was determined separately for the low and high areas (<55% and ≥55%, respectively). (b) Patch areas from (a) are plotted against the number of deacylation runs. Dashed lines indicate the trend.


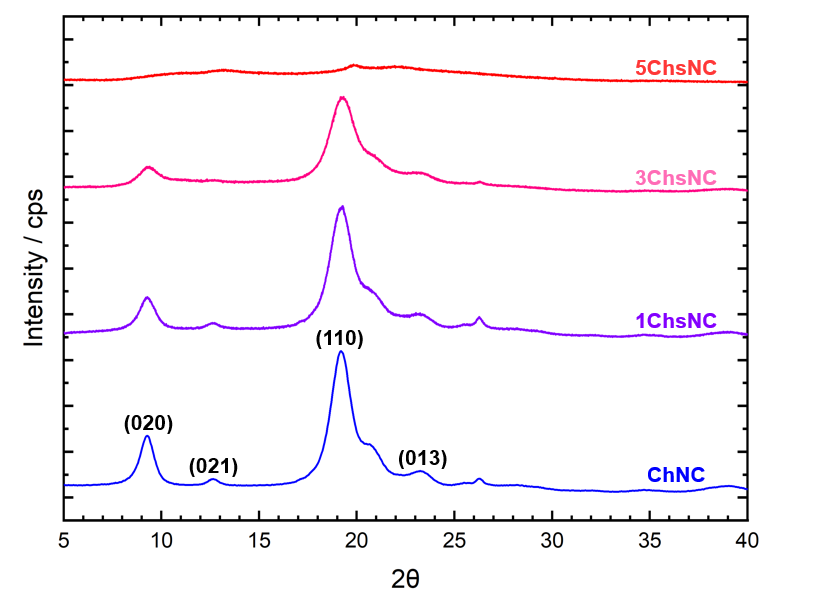


**Supplemental Figure S13:** pXRD spectra of the nanocrystal series.

**
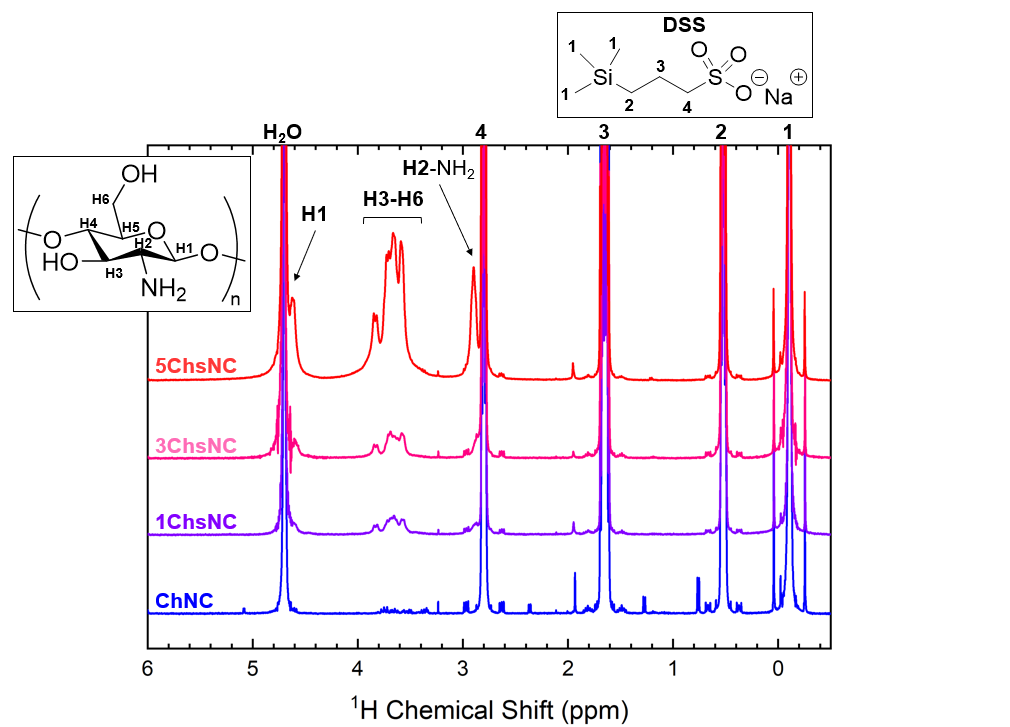
**

**Supplemental Figure S14**: ^1^H NMR spectra of the nanocrystal series, using DSS as an internal standard. The calculation for the percentage of glucosamine residues in comparison to the bulk was done via integration of the **1** peak of DSS (9 protons) compared to the integration of **H3-H6** of chitosan (5 protons).


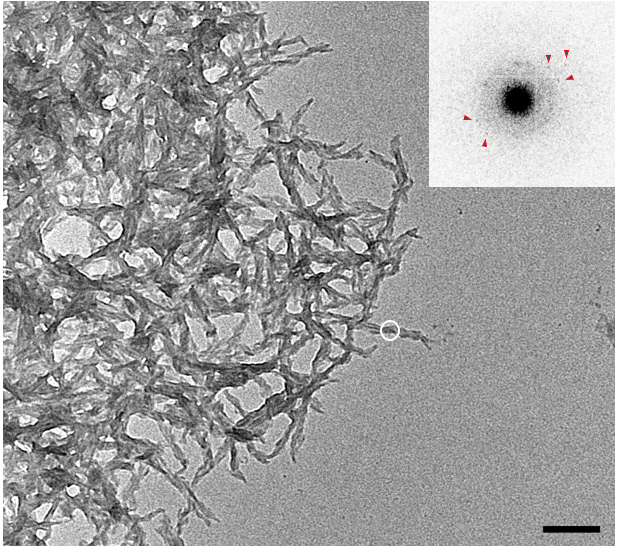


**Supplemental Figure S15**: TEM of 5ChsNC with added 0.1 M NaOH until pH 12. The white circle depicts where NBED was performed with the diffraction pattern shown in the inset.


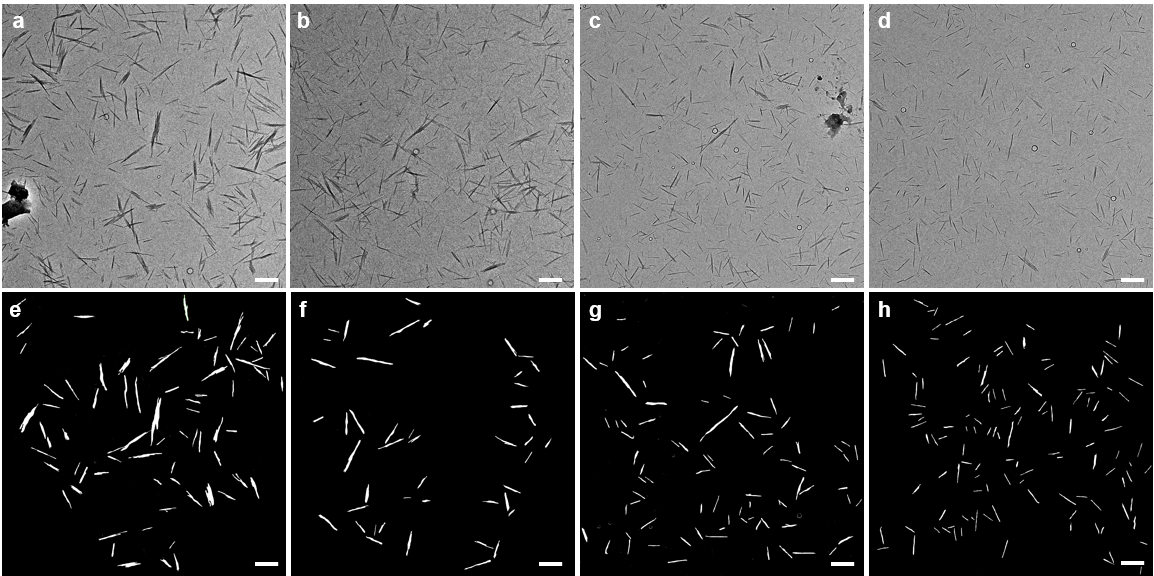


**Supplemental Figure S16:** TEM micrographs and representative tracings of (a,e) ChNC, (b,f) 1ChsNC, (c,g) 3ChsNC, (d,h) 5ChsNC. The tracings were done on ImageJ using the “Freehand selections” tool, and the Shape Filter plugin was used to calculate the shape values. Scale bars are 200 nm.


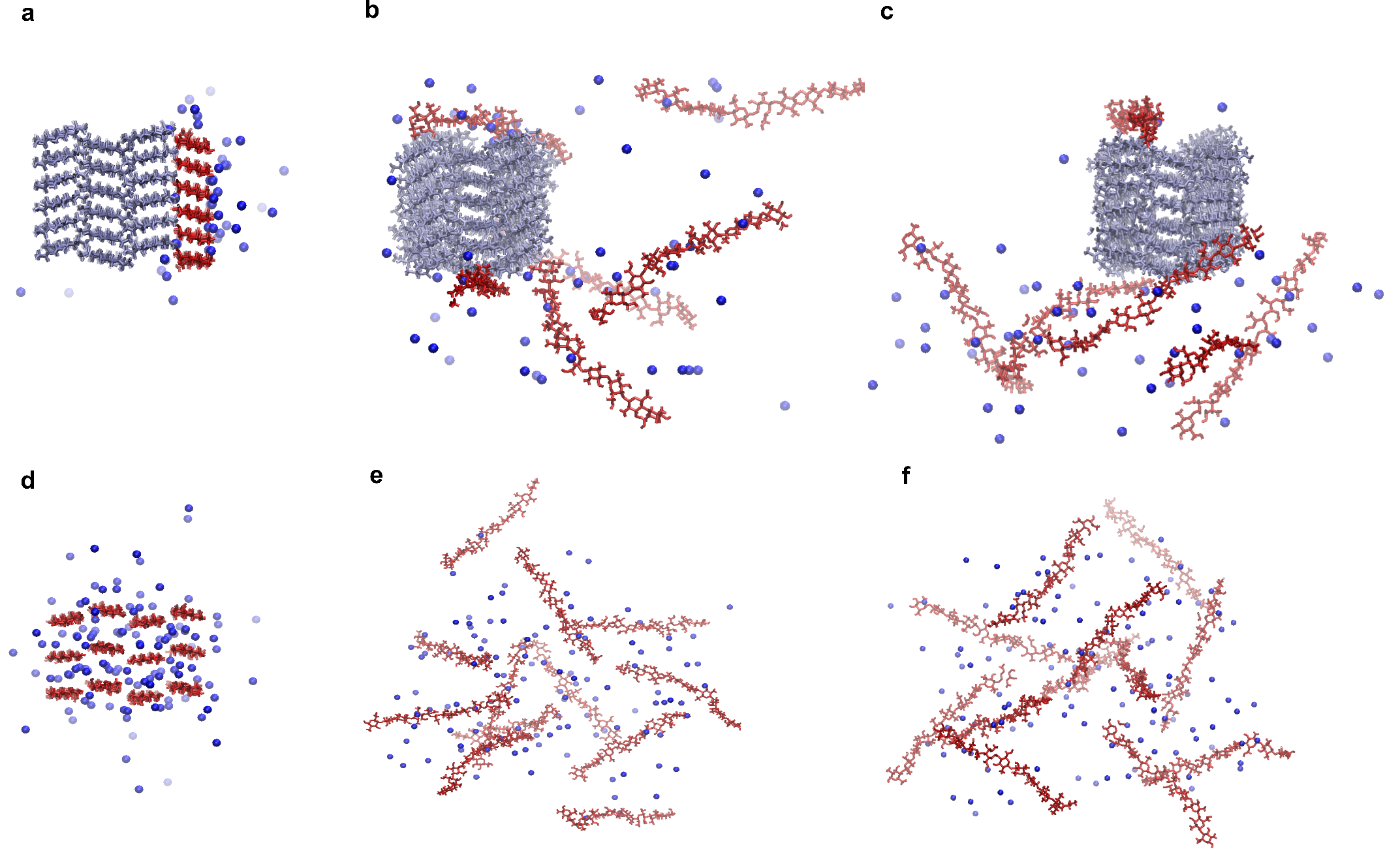


**Supplemental Figure S17:** Molecular dynamics (MD) simulations depicting (a) a model chitin crystal, where all the chains on the edge of the crystal, shown in red, are completely deacetylated (turned to chitosan). Here, the chitosan chains are protonated and Cl^-^ counter ions, shown as blue dots, are added to neutralize charges. The snapshot is taken after 20 ns equilibration in water with positional restrains on the chitin/chitosan chains. (b) The system after 50 ns and (c) 100 ns simulation in water without positional restraints. The chitosan chains are fully dissociated from the chitin crystal. (d) A model for protonated all-chitosan system, including the Cl^-^ counter ions. The snapshot is taken after 20 ns equilibration in water with positional restrains on the chitosan chains (e) The all-chitosan system after 50 ns and (f) 100 ns simulation in water without positional restrains.


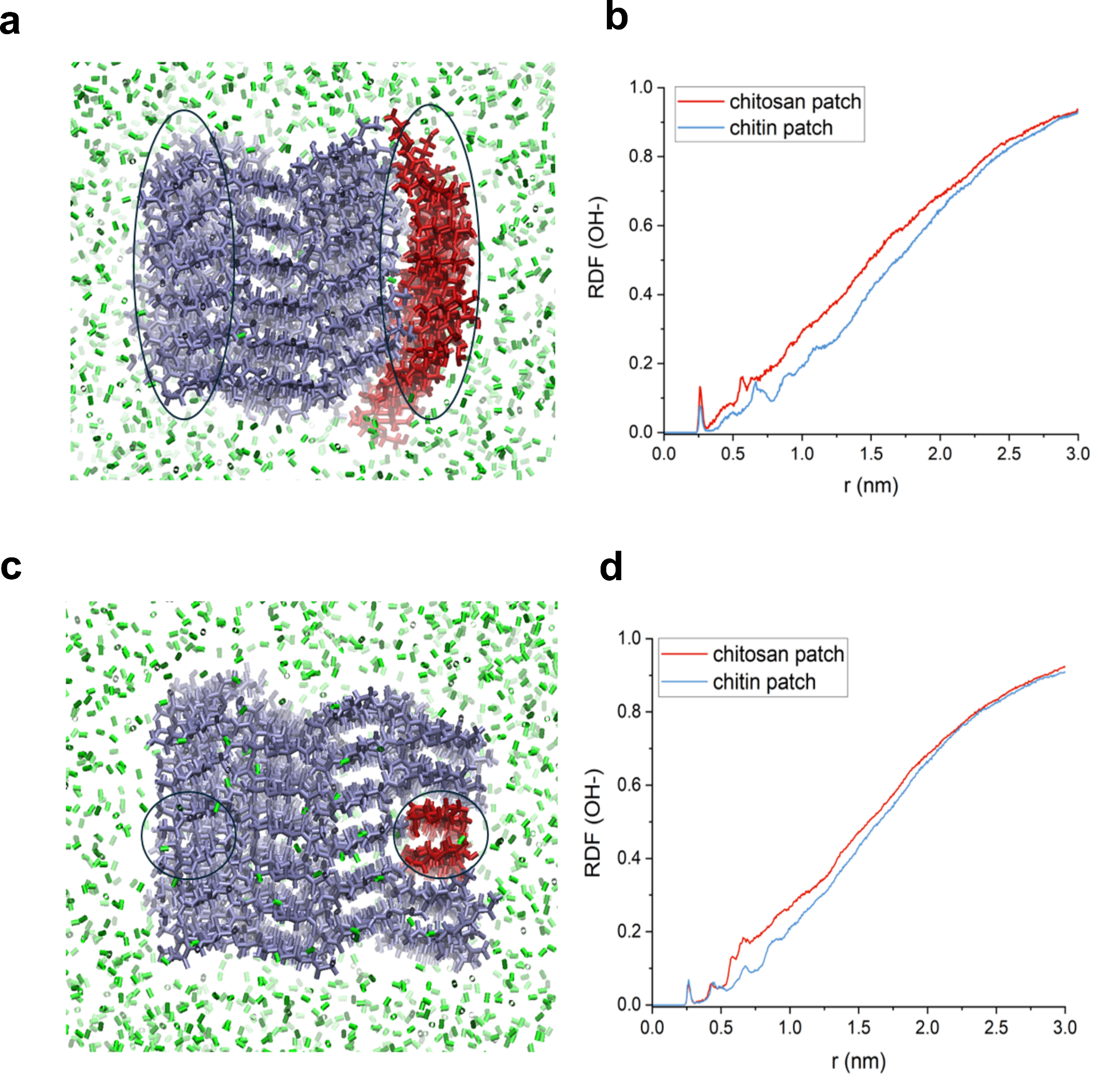


**Supplemental Figure S18:** (a) The model system shown in Figure 5.a after 200 ns simulation in NaOH 40 w/w% (OH^-^ ions are shown in green). (b) radial distribution function for OH^-^ around the chitosan patch compared to the mirror chitin patch. (c) A model system where only two chains are fully deacetylated to mimic a deacetylated patch and (d) the corresponding radial distribution function graph.

**References**

(1) Narkevicius, A.; Steiner, L. M.; Parker, R. M.; Ogawa, Y.; Frka-Petesic, B.; Vignolini, S. Controlling the Self-Assembly Behavior of Aqueous Chitin Nanocrystal Suspensions. *Biomacromolecules* **2019**, *20*, 2830-2838.

(2) Jin, T.; Kurdyla, D.; Hrapovic, S.; Leung, A. C. W.; Régnier, S.; Liu, Y.; Moores, A.; Lam, E. Carboxylated Chitosan Nanocrystals: A Synthetic Route and Application as Superior Support for Gold-Catalyzed Reactions. *Biomacromolecules* **2020**, *21*, 2236-2245.

(3) Kanevche, K.; Burr, D. J.; Nürnberg, D. J.; Hass, P. K.; Elsaesser, A.; Heberle, J. Infrared nanoscopy and tomography of intracellular structures. *Commun. Biol.* **2021**, *4*, 1341.

(4) Humphrey, W.; Dalke, A.; Schulten, K. VMD: Visual molecular dynamics. *J. Mol. Graph.* **1996**, *14*, 33-38.

(5) Case, D.; Belfon, K.; Ben-Shalom, I.; Brozell, S.; Cerutti, D.; Cheatham III, T.; Cruzeiro, V.; Darden, T.; Duke, R.; Giambasu, G. AMBER 20. *University of California: San Francisco, CA, USA* **2020**.

(6) Kirschner, K. N.; Yongye, A. B.; Tschampel, S. M.; González-Outeiriño, J.; Daniels, C. R.; Foley, B. L.; Woods, R. J. GLYCAM06: A generalizable biomolecular force field. Carbohydrates. *J. Comput. Chem.* **2008**, *29*, 622-655.

(7) Berendsen, H. J.; Grigera, J.-R.; Straatsma, T. P. The missing term in effective pair potentials. *J Phys Chem-Us* **1987**, *91*, 6269-6271.

(8) Abraham, M. J.; Murtola, T.; Schulz, R.; Páll, S.; Smith, J. C.; Hess, B.; Lindahl, E. GROMACS: High performance molecular simulations through multi-level parallelism from laptops to supercomputers. *SoftwareX* **2015**, *1-2*, 19-25.

(9) Bussi, G.; Donadio, D.; Parrinello, M. Canonical sampling through velocity rescaling. *J. Chem. Phys.* **2007**, *126*.

(10) Bernetti, M.; Bussi, G. Pressure control using stochastic cell rescaling. *J. Chem. Phys.* **2020**, *153*.
